# Supplementary material for: Patient satisfaction with hospital care and nurses in England: an observational study
Source: BMJ Open. 2018 Jan 11;8(1):e019189. doi: 10.1136/bmjopen-2017-019189 (PMC5781188; doi:10.1136/bmjopen-2017-019189)
Supplement: Supplementary table 1 [file bmjopen-2017-019189supp001.pdf]

Appendix

Patient Responses To Survey Items Related to Their Care.

Survey Question	NHS Survey Sample		RN4CAST Sample	
	Number	Percent	Number	Percent
**Overall Rating of Care**				
**Very Good, Good, Fair or Poor**	11,290	61.3	3,183	62.0
**Excellent**	7,128	38.7	1,950	38.0
**Total**	18,418	100.0	5,133	100.0
**Wanted To Complain About Your Care**				
**Yes**	1,727	9.5	489	9.7
**No**	16,422	90.5	4,554	90.3
**Total**	18,149	100.0	5,043	100.0
**Felt Treated with Respect and Dignity**				
**No or Only Sometimes**	4,400	24.3	1,254	24.3
**Always**	14,031	75.7	3,899	75.7
**Total**	18,431	100.0	5,153	100.0
**Completely Explained Purpose of Medicines***				
**No or Only to Some Extent**	3,909	28.7	1,170	30.4
**Yes Completely**	9,726	71.3	2,684	69.6
**Total**	13,635	100.0	3,854	100.0
**Doctors and Nurses Work Together Excellent**				
**Very Good, Good, Fair or Poor**	11,929	64.9	3,325	64.9
**Excellent**	6,443	35.1	1,801	35.1
**Total**	18,372	100.0	5,126	100.0
**Got Answers I Could Understand**				
**No or Only Sometimes**	6,330	37.4	1,790	37.9
**Always**	10,596	62.6	2,933	62.1
**Total**	16,926	100.0	4,723	100.0
**Always Have Confidence and Trust In Nurses**				
**No or Only Sometimes**	5,197	27.6	1,413	27.1
**Always**	13,619	72.4	3,806	72.9
**Total**	18,816	100.0	5,219	100.0
**Always Have Confidence and Trust In Doctors**				
**No or Only Sometimes**	4,636	24.7	1,294	24.9
**Always**	14,112	75.3	3,905	75.1
**Total**	18,748	100.0	5,199	100.0
**Enough Nurses on Duty to Care for You**				
**Sometimes, Rarely or Never**	8,343	44.6	2,363	45.5
**Always or Nearly Always**	10,378	55.4	2,832	54.5
**Total**	18,721	100.0	5,195	100.0

Note: Survey items for which differences are significant (at p < .05) are denoted by asterisks.
