# Supplementary material for: Patient satisfaction with hospital care and nurses in England: an observational study
Source: BMJ Open. 2018 Jan 11;8(1):e019189. doi: 10.1136/bmjopen-2017-019189 (PMC5781188; doi:10.1136/bmjopen-2017-019189)
Supplement: Author's manuscript [file bmjopen-2017-019189.draft_revisions.pdf]

BMJ Open is committed to open peer review. As part of this commitment we make the peer review history of every article we publish publicly available.

When an article is published we post the peer reviewers' comments and the authors' responses online. We also post the versions of the paper that were used during peer review. These are the versions that the peer review comments apply to.

The versions of the paper that follow are the versions that were submitted during the peer review process. They are not the versions of record or the final published versions. They should not be cited or distributed as the published version of this manuscript.

BMJ Open is an open access journal and the full, final, typeset and author-corrected version of record of the manuscript is available on our site with no access controls, subscription charges or pay-per-view fees (http://bmjopen.bmj.com).

If you have any questions on BMJ Open's open peer review process please email editorial.bmjopen@bmj.com

BMJ Open

Patient Satisfaction with Hospital Care and Nurses in England: An Observational Study

Journal:	*BMJ Open*
Manuscript ID	bmjopen-2017-019189
Article Type:	Research
Date Submitted by the Author:	16-Aug-2017
Complete List of Authors:	Aiken, Linda; University of Pennsylvania, School of Nursing<br>Sloane, Douglas; University of Pennsylvania, School of Nursing<br>Ball, Jane; National Institute for Health Research Collaboration for Leadership in Applied Health Research and Care (Wessex),University of Southampton, Centre for Innovation and Leadership in Health Sciences<br>Bruyneel, Luk; KU Leuven, Institute for Healthcare Policy<br>Rafferty, Anne Marie; King's College London, Florence Nightingale School of Nursing and Midwifery<br>Griffiths, Peter; University of Southampton, Faculty of Health Sciences
**Primary Subject Heading**:	Nursing
Secondary Subject Heading:	Health services research, Patient-centred medicine
Keywords:	patient satisfaction, hospitals, nurses, England, quality of health care

SCHOLARONE™ Manuscripts

TITLE PAGE

Patient Satisfaction with Hospital Care and Nurses in England: An Observational Study

Linda H Aiken (1)
Douglas M Sloane (1)
Jane Ball (2)
Luke Bruyneel (3)
Anne Marie Rafferty (4)
Peter Griffiths (2)

Correspondence to Linda H Aiken, Center for Health Outcomes and Policy Research, School of Nursing, University of Pennsylvania 418 Curie Boulevard, Philadelpha, PA, 19104 USA laiken@nursing.upenn.edu

Author affiliations

(1) University of Pennsylvania School of Nursing, Center for Health Outcomes and Policy Research, Philadelphia, Pennsylvania, USA
(2) Faculty of Health Sciences, University of Southampton, Southampton, England
(3) University of Leuven, Leuven Institute for Healthcare Policy, Leuven, Belgium
(4) King's College London, Florence Nightingale School of Nursing and Midwifery, London, England

Twitter: Follow Linda Aiken at @LindaAiken_Penn; Jane Ball at @JaneEBall; Luk Bruyneel at @LukBruyneel; Peter Griffiths at @workforcesoton

Acknowledgements: We thank Tim Cheney for analytic assistance, the patients, nurses, and hospitals that participated in our study, and the RN4CAST Consortium.

Contributors: LHA raised the funding, developed the idea for the study, interpreted results, drafted and revised manuscript; DMS conducted the analysis, drafted the results, contributed to manuscript revisions; JB collected survey data, contributed to manuscript development; LB contributed to results interpretation and manuscript drafting and revisions; AMR contributed to data collection, interpretation of results, manuscript revisions; PG contributed to obtaining data, results interpretation, manuscript development and revisions.

Funding: European Union's Seventh Framework Program (223468) and the National Institute of Nursing Research, National Institutes of Health (R01 NR014855).

Competing interests: None declared

Ethics approval: The study was approved by the University of Pennsylvania human subjects committee.

Keywords: Hospitals, Quality of Health Care, Nurses, England, Patient Satisfaction

Word Count: 3573

Data Sharing: No additional data are available.

For peer review only - http://bmjopen.bmj.com/site/about/guidelines.xhtml

Patient Satisfaction with Hospital Care and Nurses in England: An Observational Study

ABSTRACT (285 words)

Objectives –To inform healthcare workforce policy decisions by showing how patient perceptions of hospital care are associated with confidence in nurses and doctors, nurse staffing levels, and hospital work environments.

Design – Cross-sectional surveys of 66,348 hospital patients and 2,963 inpatient nurses.

Setting – Patients surveyed were discharged in 2010 from 161 National Health Service (NHS) trusts in England. Inpatient nurses were surveyed in 2010 in a sample of 46 hospitals in 31 of the same 161 trusts.

Participants - The 2010 NHS Survey of Inpatients obtained information from 50% of all patients discharged between June and August. The 2010 RN4CAST England Nurse Survey gathered information from inpatient medical and surgical nurses.

Main outcome measures – Patient ratings of their hospital care, their confidence in nurses and doctors , and other indicators of their satisfaction. Missed nursing care was treated as both an outcome measure and explanatory factor.

Results – Patients' perceptions of care are significantly eroded by lack of confidence in either nurses or doctors, and by increases in missed nursing care. The average number of types of missed care was negatively related to six of the eight outcomes-- odds ratios ranged from 0.78 (95% CI = 0.68 to 0.90) for excellent care ratings to 0.86 (95% CI = 0.77 to 0.95) for medications completely explained-- positively associated with higher patient-to-nurse ratios (b = 0.15, 95% CI = 0.10 to 0.19), and negatively associated with better work environments (b = -0.26, 95% CI = -0.48 to -0.04).

Conclusions – Confidence in nurses is of equal importance to confidence in doctors in patient perceptions of care. Patient perceptions of care are also strongly associated with missed nursing care, which in turn is related to nurse staffing and hospital work environments.

For peer review only

For peer review only - http://bmjopen.bmj.com/site/about/guidelines.xhtml

Strengths and limitations of this study

- This is the first quantitative study to determine the association between patients' confidence in nurses and doctors, RN staffing, and patient experiences with hospital care in NHS hospitals in England using the national NHS Adult Inpatient Survey.
- Unique data previously unavailable enable a rigorous analysis of patient to RN staffing ratios, missed nursing care, and patient satisfaction with hospital care.
- The study uses cross-sectional data, and while a number of alternative explanations are considered in our models, we cannot rule out the possibility that omitted variables contribute to associations found.

For peer review only

For peer review only - http://bmjopen.bmj.com/site/about/guidelines.xhtml

Patient Satisfaction with Hospital Care and Nurses in England: An Observational Study

INTRODUCTION

Highly publicized reports citing preventable deaths and deficiencies in hospital care in England have uniformly concluded that inadequate hospital nurse (RN) staffing is a contributing factor (1-3). Studies confirm large variation in patient to RN ratios across National Health Service (NHS) hospitals, and this variation is associated with higher mortality in hospitals where RNs care for more patients each (4-6). However, despite national guidance on safe nurse staffing (7), substantial variation still exists and the value of higher RN staffing levels is still questioned at the policy level (8). Recently introduced NHS workforce initiatives have been framed in the unsubtantiated narrative that quality deficiences in hospitals are due to "uncaring" nurses (9,10). The National Advisory Group on the Safety of Patients in England specifically advised that nurses and other NHS staff not be blamed for quality deficits, pointing instead to the need to address insufficient RN staffing (3). Nevertheless new workforce initiatives have been introduced by the NHS purportedly to produce more caring nurses. One such initiative creates a new provider category, the nursing associate, with substantially lower qualifications than RNs (11). Adding lesser trained providers to the hospital workforce without adding more RNs results in eroding the nursing skill mix that evidence suggests is associated with higher mortality and lower patient satisfaction (12). Also, the NHS is reinstating apprendice training for RNs (13), in direct opposition to a major recommendation of the 2010 Prime Minister's Commission on the Future of Nursing and Midwifery in England (14) that all nursing education should take place in universities because evidence shows that hospitals with a higher proportion of bachelors-prepared nurses have signficiantly better patient outcomes (6,15,16).

For peer review only - http://bmjopen.bmj.com/site/about/guidelines.xhtml

The concern about nurses being uncaring or lacking in compassion, and subsequent NHS nursing initiatives, have come about largely in response to case studies of poor care in a relatively small number of NHS trusts and anecedoctal reports of patient dissatisfaction. Surprisingly little use has been made of the NHS National Inpatient Survey of patients to inform strategies to improve care (17). When initiated in 2001, England's annual national survey of patients following a hospital inpatient stay was the first in the world; it aimed to make the NHS more patient-centered and more responsive to patient feedback (18). A report published in 2007 using the NHS Inpatient Survey found evidence that the experiences of staff working in the NHS mirrored the experiences of patients receiving care (19). This is a worrisome finding given the evidence showing high nurse burnout and job dissatisfaction is common in NHS hospitals (10, 20), and that 85% of RNs in NHS hospitals report not being able to complete needed nursing care due to lack of time associated with high patient to nurse workloads (21). Further, missed nursing care associated with high patient to nurse workloads is associated with an increased risk of patient mortality following common surgical procedures in nine European countries including England (22).

Studies of patients' experiences with inpatient care in the U.S., another country with mandated hospital patient satisfaction surveys, show that patients in hospitals with better RN staffing report higher overall ratings of their hospitals (23, 24). Missed nursing care is associated with less favorable patient satisfaction in U.S. hospitals (25) and European hospitals but not including England (26). There are not comparable studies in England using the NHS National Inpatient Survey that could help determine whether nursing is the problem or the solution to improving patient satisfaction in hospitals.

For peer review only

For peer review only - http://bmjopen.bmj.com/site/about/guidelines.xhtml

In this report we use survey data from the NHS National Inpatient Sample to determine the extent to which patient satisfaction is a function of specific aspects of care that pertain to nurses. For a subset of hospitals participating in the National Inpatient Survey, we link patient survey results to nurse surveys in the same hospitals that provide information on actual RN staffing and adequacy of hospital work environments as well as the extent of missed nursing care due to high workloads. The paper seeks to identify an action agenda that may hold promise for improving patients' experiences with hospital care in England.

METHODS

Data sources and samples

Patient survey data are from the 2010 NHS Survey of Inpatients, which gathered information from over 66,000 patients who were discharged from 161 acute and specialist NHS trusts in England (27). Nurse survey data are from the 2010 RN4CAST-England study (28, 20) which gathered information from 2,963 inpatient medical and surgical direct care RNs in a representative sample of 31 of the same 161 NHS trusts. These 31 trusts comprise 46 different hospitals from which 12,581 of the 66,348 patients surveyed were discharged. Of these 12,851 patients, 5,311 were in general medicine or general surgery wards. The sample of hospitals in which nurses were surveyed, described elsewhere in detail (21, 28), was a stratified random sample selected to include teaching and non-teaching hospitals of different sizes in every geographic region of England. There are no remarkable differences between the sample of hospitals in which nurses were surveyed and the other hospitals participating in the NHS Survey of Inpatients, nor were there any differences in patient characteristics or responses between the full NHS survey and the 31 trusts studied, as noted in the Appendix table. The response rate for

For peer review only - http://bmjopen.bmj.com/site/about/guidelines.xhtml

the NHS patient survey was 50%. The response rate for the nurse survey was 37%. The nurse survey has good established predictive validity in previous research (6, 29), showing, for example, that nurses' reports of quality of care are closely associated with patient mortality derived from independent data sources (30).

Patients were not participants in the initial design of the overall study but were actively engaged in the development of measures of patients' experiences with care used in the study. The Picker Institute, developers of the NHS Adult Inpatient Survey, employed patient focus groups and cognitive interviews with patients during pilot testing. Patients were offered one page to describe what they thought of the inpatient questionnaire and which aspects of patient care were most important to them. The qualitative research did not identify major questions missing from the survey but it did lead to minor modifications that were incorporated (31). Patients in our study are anonymous. We have a detailed plan to disseminate the study results through print, broadcast, and social media in every participating country. We gratefully acknowledge the contributions of participating patients in the acknowledgement section.

Analysis strategy

These data were used to undertake three distinct but related analyses. First, we use patient data from all 161 trusts to describe how patients rated their care, how their ratings varied depending on their perceptions of whether there were enough nurses on duty to provide needed care, and how they were as much a function of their confidence in nurses as their confidence in doctors. We then used the nurse data from the 46 hospitals in the 31 trusts to describe the variation in RN staffing and hospital work environments, and then used least-squares regression models with and without control variables to show how lower RN staffing levels and poorer

For peer review only - http://bmjopen.bmj.com/site/about/guidelines.xhtml

work environments are related to needed but missed nursing care. Finally, since patient survey data were only available at the trust level, we merged the nurse data from the 31 trusts with patient data from those same trusts and used logistic regression models to estimate whether and to what extent the overall level of missed nursing care in the different trusts affect patients' ratings of their care and their confidence in nurses, before and after controlling for potential confounds. Because the nurse survey was restricted to nurses on medical and surgical units, this final step of the analysis was restricted to patients in general surgical and medical wards (5,311 out of 12,851 patients in the study trusts).

RESULTS

Nurses, Doctors, and Patient Ratings of Care.

Table 1 and **Figure 1** use data from 66,348 patients in 161 trusts collected in the 2010 NHS Survey of Inpatients to show how patients' ratings of their care are highly associated with their confidence in nurses and in doctors, and with their perceptions of whether there were enough nurses to provide needed care. The first column of Table 1 shows the percentages of patients responding to the NHS survey that reported having confidence and trust in the nurses and doctors treating them, as well as the percentage that reported that there were enough nurses. The second column of Table 1 shows the percentages of patients that rated their care as excellent, based on how much confidence and trust they had in their nurses and doctors, and their perceptions of the adequacy of the number of nurses on duty. Similar percentages of patients always had confidence and trust in their doctors (80%) and nurses (75%), and very few patients had no trust in their doctors and nurses (3% in each case). Only 60% of the patients indicated that there were always enough nurses to care for them, and one in 10 patients indicated that there were never or rarely enough nurses. Hospital care was rated excellent by over half of the patients

For peer review only - http://bmjopen.bmj.com/site/about/guidelines.xhtml

who indicated that they always had confidence and trust in their doctors (53%) and in their nurses (55%). However, care was rated as excellent by only 10% of the patients who only sometimes had confidence and trust in their doctors or nurses, and by only 3% of the patients who never had confidence and trust in their doctors or nurses. Similarly, hospital care was rated as excellent by 57% of the patients who indicated that there were always enough nurses to care for them, while it was rated excellent by only 27% of the patients who said there were only sometimes enough nurses, and by only 14% of the patients who said there were rarely or never enough nurses.

While Table 1 makes it clear that nurses, like doctors, are importantly related to patients' perceptions of the quality of their care, Figure 1 shows more directly that confidence and trust in nurses is of equal importance to confidence and trust in doctors. Sixty percent of patients who have confidence and trust in both doctors and nurses rate their care as excellent, while only three percent of patients who have confidence and trust in neither rate their care as excellent. When confidence and trust in either group erodes, the result is virtually identical. Only 16% of the patients who have confidence and trust in their doctors but not nurses rate their care as excellent, and only 17% of the patients who have confidence and trust in their nurses but not doctors rate their care as excellent.

For peer review only

For peer review only - http://bmjopen.bmj.com/site/about/guidelines.xhtml

Table 1. Patient Reports about Nurses and Doctors, and the Percent Indicating Their Care was "Excellent," Based on Their Reports about Doctors and Nurses.

Patient Survey Question		Percent of Patients in Each Response Category	Percent of Patients in Each Response Category Indicating That Their Care was "Excellent"
Did you have confidence and trust in the doctors treating you?	Yes, always	80.4%	52.6%
	Yes, sometimes	16.4%	9.4%
	No	3.1%	3.4%
	Total	100.0%	
Did you have confidence and trust in the nurses treating you?	Yes, always	75.1%	55.3%
	Yes, sometimes	21.7%	10.5%
	No	3.2%	2.8%
	Total	100.0%	
Were there enough nurses on duty to care for you in the hospital?	Always or nearly always	60.4%	57.3%
	Sometimes	29.5%	26.7%
	Never or rarely	10.1%	14.1%
	Total	100.0%	

Source: Data are from the 2010 NHS Survey of Inpatients, which involved 66,348patients discharged from 161 trusts in England.

Note: The numbers reported exclude a small number (< 2%) of missing responses.

Nurse Staffing, Work Environments, and Missed Nursing Care

Nurse (RN) staffing was estimated for the 46 hospitals included in RN4CAST-England by average nurse workloads in each hospital on the day shift. Nurses reported how many patients they cared for on their last shift, and then responses are averaged across all nurses in each hospital working the day shift. Nurse workloads averaged 8.6 patients per RN during the day, and ranged from 5.6 patients per RN to 11.5 patients per RN across the 46 hospitals. Patient to

For peer review only - http://bmjopen.bmj.com/site/about/guidelines.xhtml

RN ratios were much higher and more variable at night so we elected to use day shift staffing only in our analyses.

Hospital work environment was measured by the Practice Environment Scale of the Nursing Work Index (PES-NWI), an extensively used survey-based measure with established reliability and validity (32-35) leading to its adoption by the National Quality Forum as a nurse sensitive quality of care indicator (36). The measure of work environment used is a composite measure formed from 5 subscales (comprised of 28 nurse survey items) measuring resource adequacy (4 items), nurse participation in hospital affairs (8 items), nursing foundations for quality care (9 items), nurse manager ability, leadership, and support of nurses (4 items), and nurse-physician relations (3 items). The staffing and resource adequacy subscale was dropped from the global measure used in the analysis because of its high correlation with the direct measure of RN staffing in the model, as in previous publications (37, 12).

What makes the variability in staffing and work environments across hospitals of considerable importance is that when RNs have high patient loads, and when RNs practice in poor work environments, necessary nursing care can be missed because of lack of time (21). Nurses in this study were asked whether any of thirteen important types of nursing care were needed but missed because of lack of time. **Figure 2** shows that while 7% of nurses reported that they lacked time to complete necessary pain management, and 11% missed treatments and procedures, much greater percentages reported lacking the time to educate patients and their families (52%) and comfort or talk with their patients (65%). More than a quarter of the nurses (27%) lacked the time to complete three or four of the types of care listed, just under one in five (19%) lacked the time to complete five or six of them, and another 19% lacked the time to complete seven or more of the 13 types of care listed.

For peer review only - http://bmjopen.bmj.com/site/about/guidelines.xhtml

Table 2 provides regression coefficients that indicate the effects of RN staffing and the hospital work environment on the average number of types of missed care, before and after controlling for various hospital characteristics (including size, technology, and location), and characteristics of nurses that may have affected their reports of missed care, including their role (primary nurse or shared responsibility for group of patients with other nurses), full-time status, years of experience, and unit type (medical, surgical, or combined). Higher nurse workloads (higher patient to RN ratios) are significantly related to higher numbers of types of missed care, while better work environments are significantly related to fewer types of missed care, both before and after adjustment.

Table 2. Regression Coefficients Indicating the Effects of Staffing and Practice Environment on Average Number of Types of Care Missed

	**Regression Coefficients [and 95% Confidence Intervals]**	
**Effect on Missed Care of -**	**Unadjusted**	**Adjusted**
**Patient to Nurse Ratio**	0.11***	0.15***
	[0.06,0.16]	[0.10,0.19]
**Practice Environment**	-0.30*	-0.26*
	[-0.55,-0.05]	[-0.48,-0.04]

Source: Data are from the 2010 RN4CAST-England study, which surveyed 2,963 inpatient medical and surgical direct care professional nurses (RNs) in a representative sample of 31 NHS trusts comprised of 46 different hospitals.

Notes: Adjusted Coefficients and confidence intervals are from regression models which control for hospital characteristics (Beds > 750, High Technology, and Location) and nurse characteristics (Nurse Role, Full-time Status, Years of Experience and Unit Type). Practice environment is measured by the PES-NWI tertile.

Single, double and triple asterisks denote coefficients that are significant at the .05, .01, and .001 levels, respectively.

Figures 3 and 4 show how much the number of tasks left undone varies as a function of RN staffing and hospital work environments, as estimated from the adjusted models. As the

For peer review only - http://bmjopen.bmj.com/site/about/guidelines.xhtml

number of patients per RN goes down, from 12 patients to 8 patients to 4 patients, the average number of types of missed care goes down, from 4.4 (out of 13) to 3.8 to 3.2. And, as hospital work environments improve, from relatively poor (lowest tertile) to average (middle tertile) to relatively good (highest tertile), the average number of types of missed care also goes down, from 4.2 to 4.0 to 3.7.

Missed Nursing Care and Patient Outcomes

The association of the number of types of missed care with patient outcomes is shown in **Table 3**. The coefficients in the table are odds ratios which indicate how much the odds on providing a positive response to the nine different dimensions of patient satisfaction go down as the average number of types of missed care goes up, both before (unadjusted) and after (adjusted) taking account patient characteristics that might affect their responses, including gender, age, length of stay, ward, number of long standing conditions, and type of admission (emergent/urgent or planned). In all cases, the odds ratios are less than one, indicating that positive patient appraisals of care decrease as the number of types of missed care increases; in six of the eight aspects of patient care rated the odds ratios are significant, and range from 0.78 to 0.86. These values indicate, for example, that in hospitals in which the number of types of missed care averaged 4.5 per nurse per shift, the odds on patients rating care as excellent and responding that the purpose of medicines were completely explained were 22% lower and 14% lower, respectively, than in hospitals in which the number of types of missed care averaged 3.5 per nurse per shift.

For peer review only - http://bmjopen.bmj.com/site/about/guidelines.xhtml

Table 3. Odds Ratios Indicating the Effect of the Trust Median Number of Types of Care Missed on Various Patient Outcomes

Effect of the Median Number of Types of Care Missed on Patient Outcomes	Odds Ratios [and 95% Confidence Intervals] Unadjusted	Adjusted
**Rate Care Excellent**	0.79^{***} [0.69,0.90]	0.78^{***} [0.68,0.90]
**Did Not Want To Complain About Care**	0.92 [0.76,1.12]	0.92 [0.77,1.11]
**Always Felt Treated with Respect and Dignity**	0.89 [0.78,1.02]	0.92 [0.81,1.06]
**Completely Explained Purpose of Medicines**	0.87^{*} [0.78,0.98]	0.86^{**} [0.77,0.95]
**Doctors and Nurses Work Together Excellent**	0.84^{**} [0.74,0.94]	0.82^{**} [0.72,0.93]
**Always Got Answers I Could Understand**	0.84^{**} [0.75,0.95]	0.83^{***} [0.76,0.91]
**Always Have Confidence and Trust In Nurses**	0.86^{*} [0.74,0.99]	0.85^{*} [0.73,0.99]
**Always or Nearly Always Enough Nurses**	0.87^{*} [0.76,0.99]	0.85^{**} [0.75,0.96]

Source: Data are from a merged file that included information from 31 NHS trusts for which both patient information (from 5,311 general medical and surgical patients included in the 2010 NHS Survey of Inpatients) and nurse information (from 2,963 medical and surgical nurses surveyed in the 2010 RN4CAST-England study) were available.

Notes: Adjusted models control for hospital characteristics(Beds > 750, High Technology, and Location) and patient characteristics that might affect responses, including gender, age, length of stay, ward, number of long standing conditions and type of admission (emergent/urgent or planned).

Single, double and triple asterisks denote coefficients that are significant at the .05, .01, and .001 levels, respectively.

For peer review only - http://bmjopen.bmj.com/site/about/guidelines.xhtml

DISCUSSION

National survey data from patients experiencing a hospitalization in an NHS hospital in England confirm that patients have a high level of trust and confidence in RNs, evidence that refutes the narrative blaming quality of care deficits in NHS hospitals on uncaring nurses. However, only 60 percent of patients indicated that there were always enough RNs to care for them, and one in 10 patients indicated that there were never or rarely enough RNs. The importance to patients of adequate RN staffing is evident in their responses; 57% of patients who indicated that there were always or nearly always enough RNs to care for them rated care as excellent, compared with only 14% of the patients who said there were rarely or never enough. These findings reinforce from patients' perspectives the importance of adequate hospital RN staffing.

Further insights into how quality of care might be improved in NHS hospitals is revealed when data from the NHS Inpatient Survey is linked with information on actual hospital RN staffing and nurses' assessments of the quality of their work environments. We found substantial variation across general acute hospitals in patient-to-nurse workloads. Nurses in some NHS hospitals are caring for twice as many patients at a time as nurses in other hospitals. Current NHS policies devolving greater autonomy to hospital management to make decisions about RN staffing may be contributing to the substantial observed variation in staffing, and have led experts to point to the need for checks and balances to minimize the risk of more quality failures linked to inadequate RN staffing (38). Our findings show that the substantial differences in RN staffing across NHS hospitals are associated with the extent to which needed nursing care is missed. The most frequently missed types of care include those that patients may readily recognize are missing--comforting and talking with patients, and teaching patients and family

For peer review only - http://bmjopen.bmj.com/site/about/guidelines.xhtml

members how to manage care following discharge. Our results are consistent with other research showing that higher patient workloads for RNs in NHS hospitals are associated with adverse patient outcomes including higher hospital morality (4, 5, 22). Initiatives such as those recently adopted in Wales (39) establishing an upper limit to how many patients nurses can safely and effectively care for holds promise for further improvements in patients' satisfaction with hospital care, and may save lives as well.

Another modifiable feature of hospital care found to be relevant to patients' perceptions of their care is the quality of the hospital work environment. In hospitals rated by nurses to have less favorable clinical work environments, needed but missed nursing care is more extensive. Patients' perceptions of care are less favorable when missed care is more extensive. Research suggests that hospital work environments that support RNs to provide care efficiently and effectively, and without constant interruptions because of operational failures such as missing medications and equipment (40), are reasonably low cost interventions and return good value in terms of better patient outcomes at the same or lower costs (41,42). Magnet hospitals formally recognized for their good hospital work environments have significantly higher patient satisfaction than matched non-Magnet hospitals (43). One of the first Magnet hospitals accredited outside the U.S. was a NHS trust in England, which research showed significantly improved its work environment and care quality during the process of achieving Magnet accreditation (44). Unfortunately, the NHS merged the Magnet facility out of existence after a year, and there has not been a Magnet accredited hospital in England in over 15 years.

Patients' confidence in both doctors and nurses is equally important in how patients rate their hospitals; few patients that have high confidence in their doctors but little confidence in their nurses rate their hospitals highly. This finding is relevant to policy decisions governing the

For peer review only - http://bmjopen.bmj.com/site/about/guidelines.xhtml

composition of the NHS workforce in England. Between 2010 and 2015, the number of physician consultants (mostly inpatient physicians) increased by more than one-fifth while the number of RNs increased only one percent (45).

Our study has many strengths including use of validated measures of patient satisfaction, nurse staffing, hospital work environment, and missed nursing care across large numbers of NHS hospitals. The study has limitations as well. Data from both patients and nurses is cross sectional thus limiting causal inferences about the associations found. We take into account, to the extent possible, alternative explanations about factors that could be associated with our findings including characteristics of hospitals such as teaching status, and characteristics of the patients responding to the national survey, including their health status since self-reported limiting long-term conditions have been found to be associated with less favorable perceptions of care (46). Our data are from 2010 but remain the only comprehensive data on hospital nurse workforce and patient satisfaction across large numbers of NHS hospitals in England. Moreover, our interest is in the relationship between patient satisfaction and nurse resources, and there is no reason to expect the relationship to have changed since 2010. Indeed, Sir Robert Francis, author of the public inquiry into quality of care deficiencies at the Mid Staffordshire NHS Trust (1), commented as recently as July 2017 that safe nurse staffing in England still lacks a standardized approach and substantial variation across hospitals in nurse staffing remains (47).

CONCLUSIONS

Patients express a high level of confidence and trust in nurses, and their satisfaction with hospital care is less favorable when they perceive there are not enough nurses available. The narrative that quality deficits in hospitals are due to “uncaring” nurses is not supported by the evidence. To the contrary, our findings suggest that ensuring adequate numbers of RNs at the

For peer review only - http://bmjopen.bmj.com/site/about/guidelines.xhtml

hospital bedside and improved hospital clinical care environments are promising strategies for enhancing patient satisfaction with care.

For peer review only

For peer review only - http://bmjopen.bmj.com/site/about/guidelines.xhtml

Acknowledgements: We thank Tim Cheney for analytic assistance, the patients, nurses, and hospitals that participated in our study, and the RN4CAST Consortium.

Competing interests: None declared

Funding: European Union's Seventh Framework Program (223468) and the National Institute of Nursing Research, National Institutes of Health (R01NR014855).

For peer review only

For peer review only - http://bmjopen.bmj.com/site/about/guidelines.xhtml

REFERENCES

1. Francis R. Report of the Mid Staffordshire NHS Foundation Trust Public Inquiry; 2013. Available from: http://webarchive.nationalarchives.gov.uk/20150407084003/http://www.midstaffspublicinquiry.com/report.
2. Keogh B. Review into the quality of care and treatment provided by 14 hospital trusts in England: overview report; 2013. Available from: http://www.nhs.uk/NHSEngland/bruce-keogh-review/Documents/outcomes/keogh-review-final-report.pdf.
3. National Advisory Board on the Safety of Patients in England. A promise to learn–a commitment to act: improving the safety of patients in England. London: Department of Health; 2013. Available from: www.health.org.uk/government/publications/berwick-review.
4. Rafferty AM, Clarke SP, Coles J, Ball J, James P, McKee M, et al. Outcomes of variation in hospital nurse staffing in English hospitals: cross-sectional analysis of survey data and discharge records. Int J Nurs Stud. 2007;44(2):175-82.
5. Griffiths P, Ball J, Murrells T, Jones S, Rafferty AM. Registered nurse, healthcare support worker, medical staffing levels and mortality in English hospital trusts: a cross-sectional study. BMJ Open. 2016;6(2):e008751.
6. Aiken LH, Sloane DM, Bruyneel L, Van den Heede K, Griffiths P, Busse R, et al. Nurse staffing and education and hospital mortality in nine European countries: a retrospective observational study. Lancet. 2114;383;1824-30.
7. National Institute for Health and Care Excellence (NICE). NHS England asks NICE to suspend safe staffing programme. United Kingdom: NICE, 2015. Available from: https://www.nice.org.uk/news/article/nhs-england-asks-nice-to-suspend-safe-staffing-programme.
8. Lord Carter of Coles. Operational productivity and performance in English NHS acute hospitals: unwarranted variation. February 2016. Available from: https://www.gov.uk/government/uploads/system/uploads/attachment_data/file/498107/Carter_Review_-_executive_summary.pdf.
9. Smith R. Nurses lack compassion NHS admits. The Telegraph. 2012 January 18.
10. Aiken L, Rafferty AM, Sermeus W. Caring nurses hit by a quality storm. Nursing Standard. 2014;28(35):22-5.
11. Gummer B. Nursing associate role offers new route into nursing. UK: Department of Health. [cited 2016 Jan 25]. Available from: http://www.gov.uk/government/news/nursing-associate-role-offers-new-route-into-nursing.
12. Aiken LH, Sloane D, Griffiths P, Rafferty AM, Bruyneel L, McHugh M, et al. Nursing skill mix in European hospitals: cross-sectional study of the association with mortality, patient ratings, and quality of care. BMJ Quality & Safety. 2016. Epub 2016 Nov 15.
13. Department of Health and Human Services. New nursing degree apprenticeship United Kingdon: GOV.UK; 2016. Available from: https://www.gov.uk/government/news/new-nursing-degree-apprenticeship.
14. Prime Minister's Commission on the Future of Nursing and Midwifery in England. Front Line. 2010. Available from: http://webarchives.gov.uk/20100331110913.
15. Aiken LH, Clarke SP, Cheung RB, Sloane DM, Silber JH. Educational levels of hospital nurses and surgical patient mortality. JAMA. 2003; 290(12);1617-1623.
16. Yakusheva O, Lindrooth R, Weiss M. Economic evaluation of the 80% baccalaureate nurse workforce recommendation.: a patient level analysis. Medical Care. 2014;52;864-869.

For peer review only - http://bmjopen.bmj.com/site/about/guidelines.xhtml

17. Coulter A, Locock L, Ziebland S, Calabrese J. Collecting data on patient experience is not enough: they must be used to improve care. BMJ. 2014;348:g2225.
18. DeCourcy A, West E, Barron D. The National Adult Inpatient Survey conducted in the English National Health Service from 2002 to 2009: how have the data been used and what do we know as a result? BMC Health Services Research. 2012;12:71.
19. Dawson J. Does the experience of staff working in the NHS link to the patient experience of care? An analysis of links between the 2007 acute trust inpatient and NHS staff surveys. Institute for Health Services Effectiveness. 2009 [cited 2017 Jun 9]. Available from: https://www.gov.uk/government/uploads/system/uploads/attachment_data/file/215457/dh_129662.pdf.
20. Aiken LH, Sloane DM, Bruyneel L, Van den Heede K, Sermeus W. Nurses' reports of working conditions and hospital quality of care in 12 countries in Europe. Int J Nurs Stud. 2013;50(2):143-53.
21. Ball JE, Murrells T, Rafferty AM, Morrow E, Griffiths P. 'Care left undone' during nursing shifts: associations with workload and perceived quality of care. BMJ Quality & Safety. 2014 Feb;23(2):116-25.
22. Ball J, Bruyneel L, Aiken LH, Sermeus W, Sloane DM, Rafferty AM, Lindqvist R, Tishelman C, Griffiths P. Post-operative mortality, missed care and nurse staffing in nine countries: a cross sectional study. Int J Nurs Studies. In press 2017.
23. Jha AK, Orav EJ, Zheng J, Epstein AM. Patients' perception of hospital care in the United States. N Engl J Med. 2008;359(18):1921-31.
24. Kutney-Lee A, McHugh MD, Sloane DM, Cimiotti JP, Flynn L, Neff DF, Aiken LH. Nursing: A key to patient satisfaction. Heath Affairs. 2009; 28(4);w669-677.
25. Lake ET, Germack HD, Viscardi MR. Missed nursing care is linked to patient satisfaction: a cross sectional study of U.S. hospitals. BMJ Quality and Safety. 2016;25:535-543.
26. Bruyneel L, Sloane DM, Smith HL, Lesaffre E, Sermeus W, Aiken LH. Organization of hospital nursing, provision of nursing care, and patient assessments of care in Europe. Medical Care Research and Review. 2015;72(6):643-664.
27. Care Quality Commission. National NHS patient survey programme Survey of adult inpatients 2010 England: NHS; 2011. Available from: http://www.nhssurveys.org/survey/1017.
28. Sermeus W, Aiken LH, Van den Heede K, Rafferty AM, Griffiths P, Moreno-Casbas MT, et al. Nurse forecasting in Europe (RN4CAST): rationale, design and methodology. BMC Nurs. 2011;10(1):6.
29. Aiken LH, Sermeus W, Van den Heede K, Sloane DM et al. Patient safety, satisfaction, and quality of care: cross-sectional surveys of nurses and patients in 12 countries in Europe and the United States. BMJ. 2012;344;e1717.
30. Smeds-Alenius L, Tishelman C, Lindqvist R, Runesdotter S, McHugh MD. RN assessments of excellent quality of care and patient safety are associated with significantly lower odds of 30-day inpatient mortality: A national cross-sectional study of acute-care hospitals. Int J Nurs Stud. 2016;61:117-124.
31. Reeves R, Coulter A, Jenkinson C, Cartwright J, Bruster S, Richards N: Development and pilot testing of questionnaires for use in the acute NHS trust inpatient survey programme. Europe: Picker Institute [cited 2016 Jan 29]. Accessible from: http://www.nhssurveys.org/Filestore/documents/DevelopmentInpatientQuestionnaire.pdf.

For peer review only - http://bmjopen.bmj.com/site/about/guidelines.xhtml

32. Lake ET. Development of the Practice Environment Scale of the Nursing Work Index. Res Nurs Health. 2002;25(3):176-88.
33. Lake ET. The nursing practice environment: measurement and evidence. Med Care Res Rev. 2007;64(2 Suppl):104S-22S.
34. Aiken LH, Clarke SP, Sloane DM, Lake ET, Cheney T. Effects of hospital care environment on patient mortality and nurse outcomes. J Nurs Adm. 2008;38(5):223-9.
35. Swiger PA, Patrician PA Miltner RS, Raju D, et al. The Practice Environment Scale of the Nursing Work Index: updated review and recommendations for use. Int J Nurs Studies. 2017;74:76-84.
36. National Quality Forum. National Voluntary Consensus Standards for Nursing-Sensitive Care: An initial performance measure set - A consensus report. Washington D.C.; 2004. Report No.: NQFCR-08-04.
37. Aiken LH, Cimiotti J, Sloane DM, Smith HL, Flynn L, Neff D. The effects of nurse staffing and nurse education on patient deaths in hospitals with different nurse work environments. Medical Care. 2011;49(12):1047-1053.
38. Buchan J, Seccombe I, Gershlick B, Charlesworth A. In short supply: pay policy and nurse numbers. London: The Health Foundation, 2017 [updated April 2017]. Available from: http://www.health.org.uk/sites/health/files/Workforce%20pressure%20points%202017%20FINAL_0.pdf.
39. National Assembly for Wales. Nurse Staffing Levels (Wales) Act 2016, United Kingdom. 2016. Accessed from: http://legislation.data.gov.uk/anaw/2016/5/data.htm?wrap=true.
40. Tucker AL, Spear SJ. Operational failures and interruptions in hospital nursing. Health Services Research 2006;41:643-662.
41. Silber J, Rosenbaum P, McHugh M, Ludwig J, Smith H, Niknam B, et al. Comparison of the value of nursing work environments in hospitals across different levels of patient risk. JAMA Surgery. 2016;151(6):527-36.
42. Kutney-Lee A, Stimpfel AW, Sloane DM, Cimiotti JP, Quinn LW, Aiken LH. Changes in patient and nurse outcomes associated with Magnet hospital recognition. Med Care. 2015;53(6):550-7.
43. Stimpfel AW, Sloane DM, McHugh MD, Aiken LH. Hospitals known for nursing excellence associated with better hospital experiences for patients. Health Serv Res. 2015;51(3):1120-34.
44. Aiken LH, Buchan J, Ball J, Rafferty AM. Transformative impact of Magnet designation: England case sudy. J Clin Nurs. 2008;17(24);3330-3337.
45. Lafond S, Charlesworth A, Roberts A. A year of plenty? An analysis of NHS finances and consultant productivity, United Kingdom; 2017 [cited 2017 June 9]. Available from: www.health.org.uk/publication/year-of-plenty.
46. Hewitson P, Skew A, Graham C, Jenkinson C, Coulter A. People with limiting long-term conditions report poorer experiences and more problems with hospital care. BMC Health Services Research. 2014;14(1):33.44.
47. Safe staffing still lacks standardized approach, warns Francis. Nursing Times. July 6, 2017. Available from: https://www.nursingtimes.net/7019357article?utm_source=newslettertutm_medium=email&utm.

For peer review only - http://bmjopen.bmj.com/site/about/guidelines.xhtml

Figure 1. Percent of Patients Rating Their Care Excellent, by Confidence in Nurses and Doctors

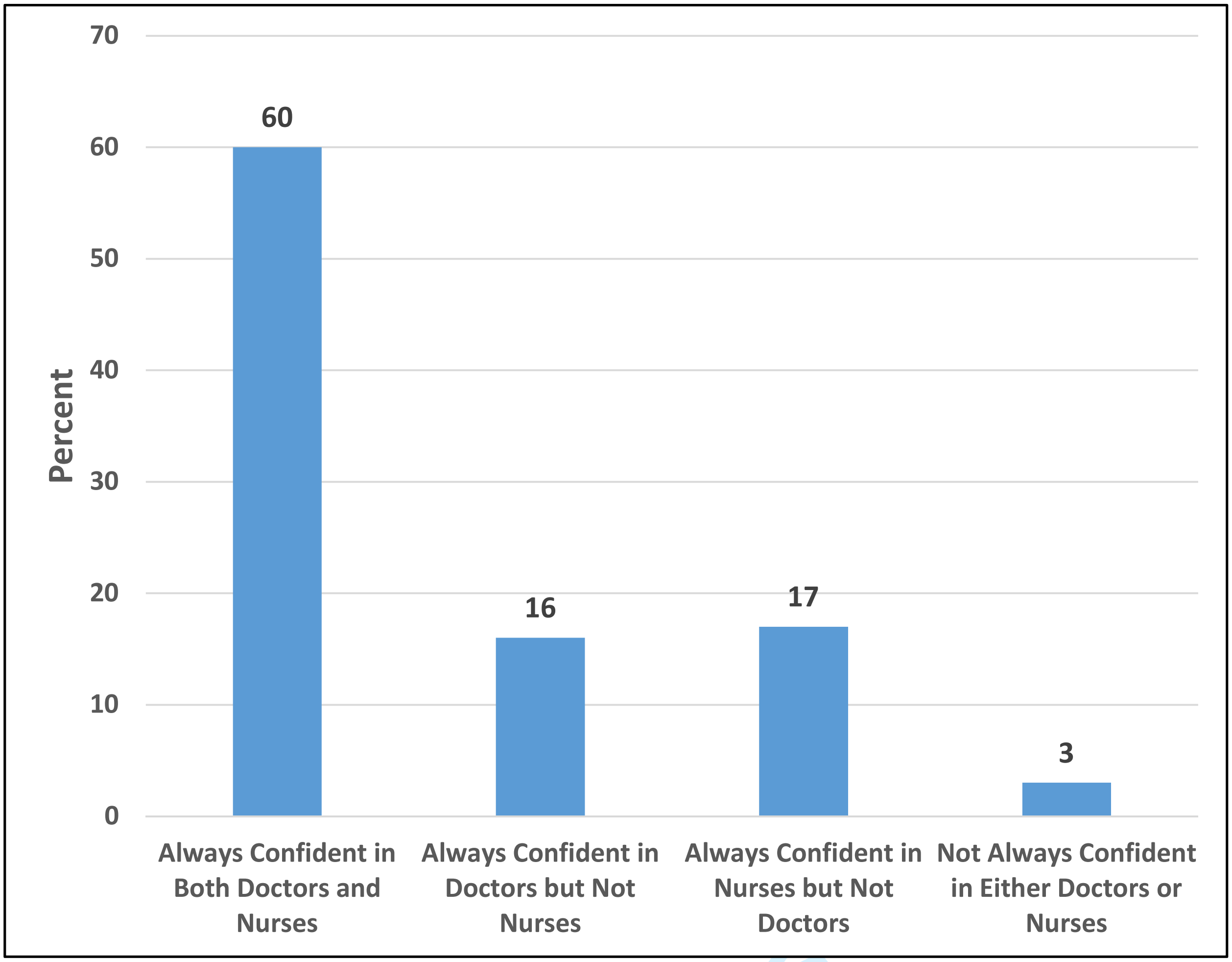

Source: Data are from the 2010 NHS Survey of Inpatients, which involved 66,348 patients discharged from 161 trusts in England.

For peer review only - http://bmjopen.bmj.com/site/about/guidelines.xhtml

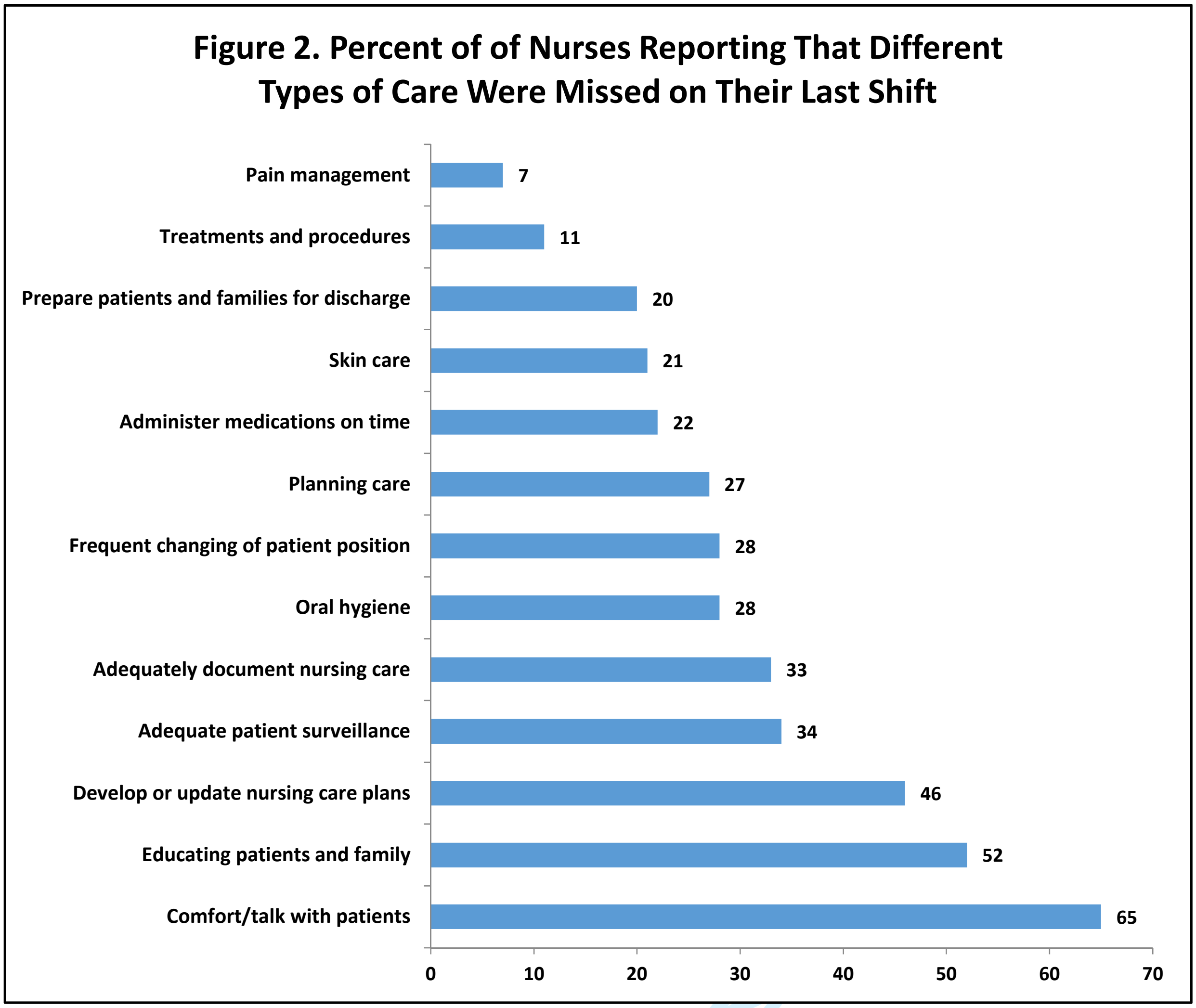

Source: Data are from the 2010 RN4CAST-England study, which surveyed 2,963 inpatient medical and surgical direct care professional nurses (RNs) in a representative sample of 31 NHS trusts comprised of 46 different hospitals.

For peer review only - http://bmjopen.bmj.com/site/about/guidelines.xhtml

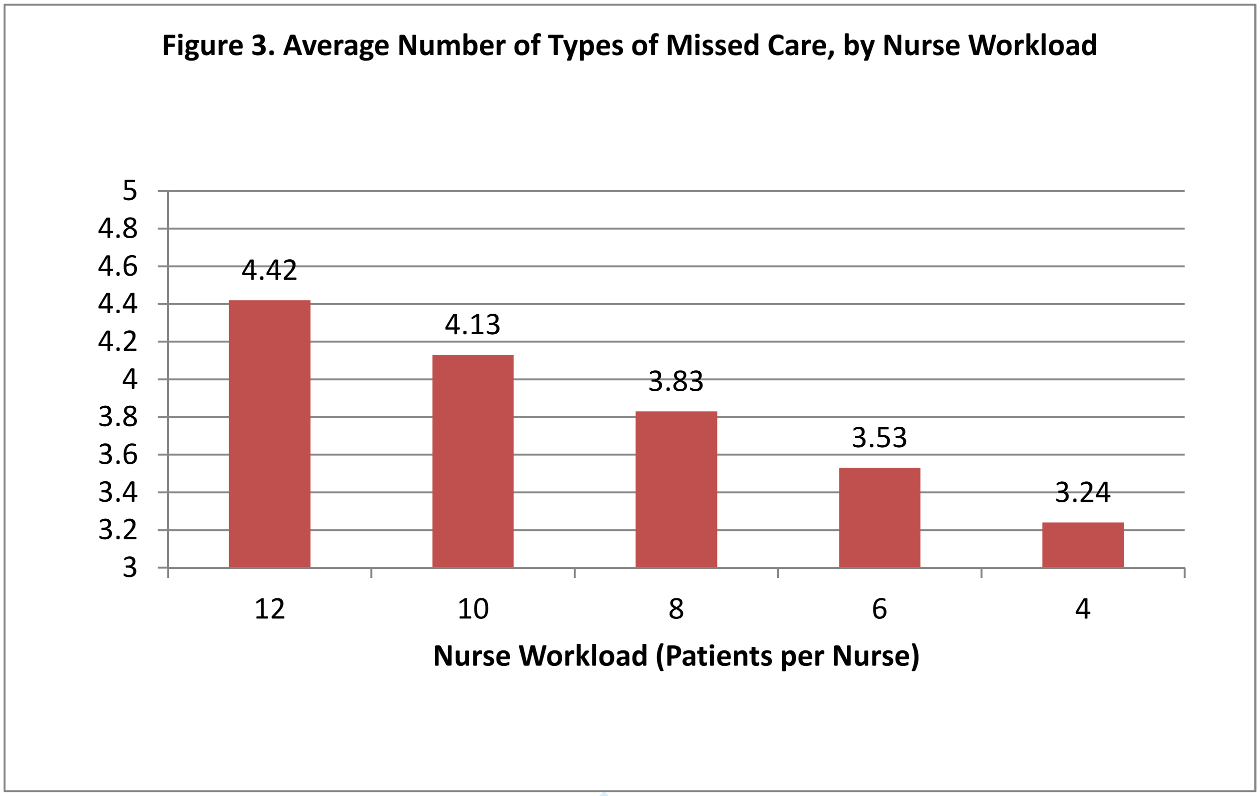

Source: Data are from the 2010 RN4CAST-England study, which surveyed 2,963 inpatient medical and surgical direct care professional nurses (RNs) in a representative sample of 31 NHS trusts comprised of 46 different hospitals.

For peer review only - http://bmjopen.bmj.com/site/about/guidelines.xhtml

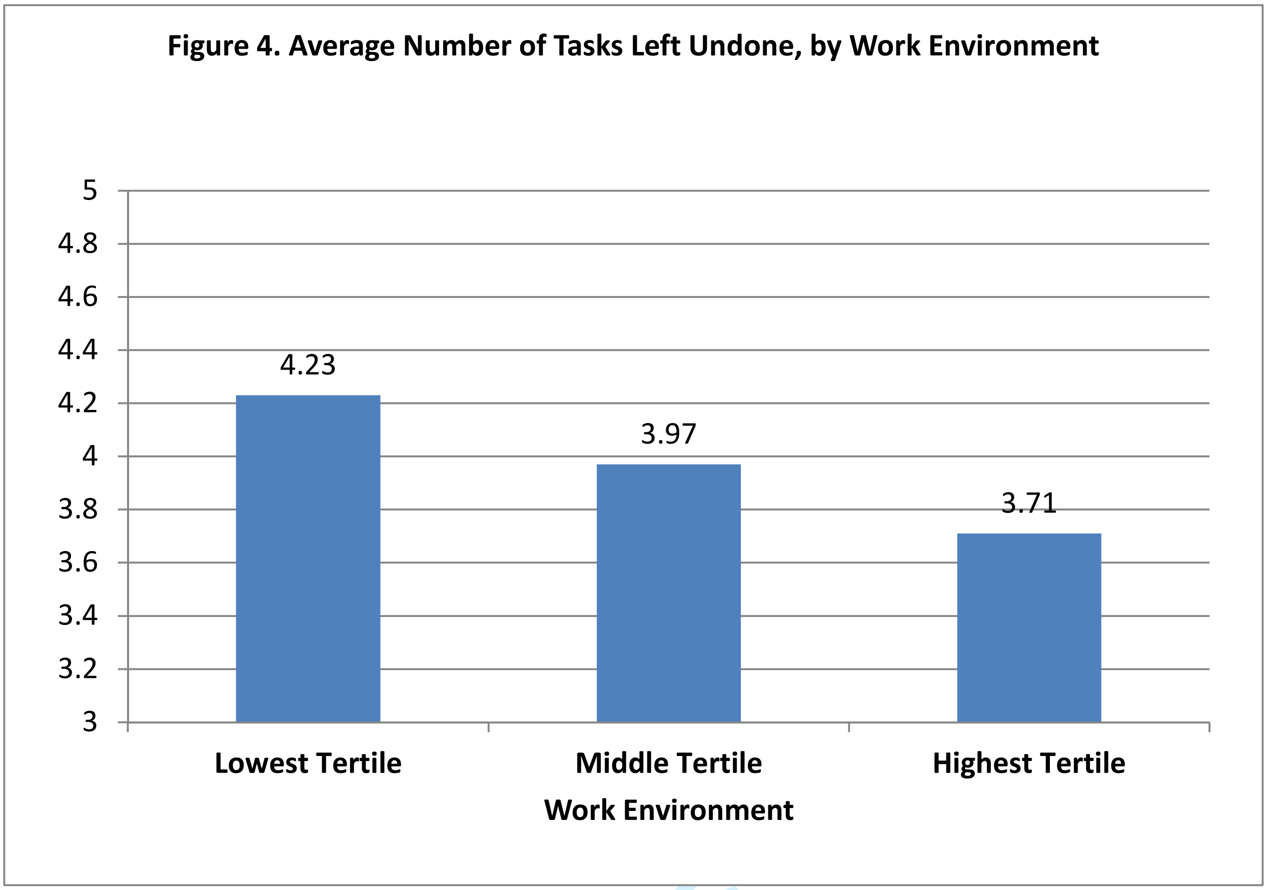

Source: Data are from the 2010 RN4CAST-England study, which surveyed 2,963 inpatient medical and surgical direct care professional nurses (RNs) in a representative sample of 31 NHS trusts comprised of 46 different hospitals.

For peer review only - http://bmjopen.bmj.com/site/about/guidelines.xhtml

Appendix

Patient Responses To Survey Items Related to Their Care.

Survey Question	NHS Survey Sample Number	NHS Survey Sample Percent	RN4CAST Sample Number	RN4CAST Sample Percent
**Overall Rating of Care**				
Very Good, Good, Fair or Poor	11,290	61.3	3,183	62.0
Excellent	7,128	38.7	1,950	38.0
Total	18,418	100.0	5,133	100.0
**Wanted To Complain About Your Care**				
Yes	1,727	9.5	489	9.7
No	16,422	90.5	4,554	90.3
Total	18,149	100.0	5,043	100.0
**Felt Treated with Respect and Dignity**				
No or Only Sometimes	4,400	24.3	1,254	24.3
Always	14,031	75.7	3,899	75.7
Total	18,431	100.0	5,153	100.0
**Completely Explained Purpose of Medicines***				
No or Only to Some Extent	3,909	28.7	1,170	30.4
Yes Completely	9,726	71.3	2,684	69.6
Total	13,635	100.0	3,854	100.0
**Doctors and Nurses Work Together Excellent**				
Very Good, Good, Fair or Poor	11,929	64.9	3,325	64.9
Excellent	6,443	35.1	1,801	35.1
Total	18,372	100.0	5,126	100.0
**Got Answers I Could Understand**				
No or Only Sometimes	6,330	37.4	1,790	37.9
Always	10,596	62.6	2,933	62.1
Total	16,926	100.0	4,723	100.0
**Always Have Confidence and Trust In Nurses**				
No or Only Sometimes	5,197	27.6	1,413	27.1
Always	13,619	72.4	3,806	72.9
Total	18,816	100.0	5,219	100.0
**Always Have Confidence and Trust In Doctors**				
No or Only Sometimes	4,636	24.7	1,294	24.9
Always	14,112	75.3	3,905	75.1
Total	18,748	100.0	5,199	100.0
**Enough Nurses on Duty to Care for You**				
Sometimes, Rarely or Never	8,343	44.6	2,363	45.5
Always or Nearly Always	10,378	55.4	2,832	54.5
Total	18,721	100.0	5,195	100.0

Note: Survey items for which differences are significant (at $p < .05$) are denoted by asterisks.

For peer review only - http://bmjopen.bmj.com/site/about/guidelines.xhtml

STROBE Statement—Checklist of items that should be included in reports of ***cross-sectional studies*** (***Note: page numbers below refer to the page numbers at the bottom of each page in the document, not the electronic page numbers).***

	Item No	Recommendation
**Title and abstract**	1	(*a*) Indicate the study's design with a commonly used term in the title or the abstract ***The observational and cross-sectional study design is noted in the Title Page (p. i) and Abstract page (p. ii, Title and Design sections).***
		(*b*) Provide in the abstract an informative and balanced summary of what was done and what was found ***The study design, settings, participants, outcome measure and results are described in the Abstract (p. ii).***
**Introduction**		
Background/rationale	2	Explain the scientific background and rationale for the investigation being reported ***The background and rationale are explained in the "Introduction" (p. 1-2)***
Objectives	3	State specific objectives, including any prespecified hypotheses ***The primary objective of the study is stated in the last paragraph of the introduction (p. 2)***
**Methods**		
Study design	4	Present key elements of study design early in the paper. ***Key elements of the study design are described in the Abstract (pg. ii) and in the Methods section of the paper (p. 3-4)***
Setting	5	Describe the setting, locations, and relevant dates, including periods of recruitment, exposure, follow-up, and data collection. ***The setting, locations and dates of the trusts, patients and nurses from which data were collected are given in the first paragraph of the Methods section (p. 3).***
Participants	6	(*a*) Give the eligibility criteria, and the sources and methods of selection of participants. ***We describe the sampling of trusts, patients and nurses in the Methods section (p. 3). We also reference prior publications which contain additional details in the text on that page.***
Variables	7	Clearly define all outcomes, exposures, predictors, potential confounders, and effect modifiers. Give diagnostic criteria, if applicable. ***Key patient variables (including patient ratings of care and satisfaction, and nurse staffing, work environments, and missed nursing car)e are described in general on p. 5-6, and additional details on control variables used are given in the note below Table 2 (p. 9).***
Data sources/ measurement	8*	For each variable of interest, give sources of data and details of methods of assessment (measurement). Describe comparability of assessment methods if there is more than one group. ***Data are from the NHS patient satisfaction survey and RN4CAST nurse survey as described on p. 3.***
Bias	9	Describe any efforts to address potential sources of bias ***We attempted to obtain unbiased results by using logistic regression models before and after adjusting for hospital, nurse and patients characteristics, as indicated in the text and notes related to Tables 2 (p. 9) and 3 (p. 11).***
Study size	10	Explain how the study size was arrived at ***We describe the sampling of trusts, patients and nurses in the Methods section (p. 3). We also reference prior publications which contain additional details in the text on p. 3.***

For peer review only - http://bmjopen.bmj.com/site/about/guidelines.xhtml

Quantitative variables	11	Explain how quantitative variables were handled in the analyses. If applicable, describe which groupings were chosen and why. ***Some quantitative variables (i.e. nurse staffing, nurse experience, number of types of missed care) were treated as continuous and not grouped. We describe grouping of work environment in the Methods section (p. 3). Numerous control variables were dichotomous and represented by dummy variables [see notes to Tables 2 (p. 9) and 3 (p. 11) and related text.***
Statistical methods	12	(*a*) Describe all statistical methods, including those used to control for confounding ***We discuss the least squares and logistic regression models we used in the Analysis Strategy sub-section of the Methods section (p. 4).***
		(*b*) Describe any methods used to examine subgroups and interactions ***We have not, as yet, investigated subgroup differences and interactions with these data.***
		(*c*) Explain how missing data were addressed ***Information on missing data for the NHS Inpatient Survey are negligible, and described in previous work that we have referenced. We not that for the measures we considered (cf., Note for Table 1, p. 7) that the numbers reported exclude a small number (< 2%) of missing responses. Missing information also was negligible for most of the measures derived from the nurse survey, and ranged from roughly 2% to 5%.***
		(*d*) If applicable, describe analytical methods taking account of sampling strategy. ***Robust regression procedures were used to account for the clustering of patients and nurses in the hospitals and trusts.***
		(*e*) Describe any sensitivity analyses ***We report unadjusted and adjusted results in Tables 2 (p. 9) and 3 (p. 11), and found that our results remained significant even after adjustment for potential confounds.***
**Results**		
Participants	13*	(a) Report numbers of individuals at each stage of study—eg numbers potentially eligible, examined for eligibility, confirmed eligible, included in the study, completing follow-up, and analysed. ***The numbers of trusts, nurses and patients sampled are given in the abstract (p. ii) and on p. 3, where response rates are indicated).***
		(b) Give reasons for non-participation at each stage. ***We note on p. 4 that nurse survey data were available for only some 31 of the 161 trusts for which***
		(c) Consider use of a flow diagram. ***Not helpful here.***
Descriptive data	14*	(a) Give characteristics of study participants (eg demographic, clinical, social) and information on exposures and potential confounders. ***Numbers and characteristics of trusts and patients are shown in Tables 1 (p. 7), 2 (p. 9), and 3 (p. 11) (Also see related text). More detailed information on the trusts, patients and nurses in the study can be found in related papers that are cited.***
		(b) Indicate number of participants with missing data for each variable of interest.
Outcome data	15*	Report numbers of outcome events or summary measures. ***The patient satisfaction outcome data is given in the Appendix (separate from Main Document).***
Main results	16	(*a*) Give unadjusted estimates and, if applicable, confounder-adjusted estimates and their precision (eg, 95% confidence interval). Make clear which confounders were adjusted for and why they were included. ***Unadjusted and adjusted estimates are***

For peer review only

For peer review only - http://bmjopen.bmj.com/site/about/guidelines.xhtml

		shown in Tables 2 (p. 9) and 3 (p. 11).
		(*b*) Report category boundaries when continuous variables were categorized. ***Category boundaries for the categorized continuous variable, nurse work environment, are described on p. 6-7 of Methods.***
		(*c*) If relevant, consider translating estimates of relative risk into absolute risk for a meaningful time period. ***Not relevant.***
Other analyses	17	Report other analyses done—eg analyses of subgroups and interactions, and sensitivity analyses ***See 12b above.***
**Discussion**		
Key results	18	Summarise key results with reference to study objectives ***(see Discussion, p. 12-14) and Conclusion p. 14-15)***
Limitations	19	Discuss limitations of the study, taking into account sources of potential bias or imprecision. Discuss both direction and magnitude of any potential bias. ***See last paragraph in Discussion section on p. 14.***
Interpretation	20	Give a cautious overall interpretation of results considering objectives, limitations, multiplicity of analyses, results from similar studies, and other relevant evidence ***See Discussion (p. 12-14) and Conclusions sections (p. 14-15).***
Generalisability	21	Discuss the generalisability (external validity) of the study results. ***While point estimates of factors (like the nursing characteristics) and satisfaction likely vary from trust to trust, we believe the associations found are likely generalizable to all NHS trusts, as we note in the conclusion at the end (p. 14-15).***
**Other information**		
Funding	22	Give the source of funding and the role of the funders for the present study and, if applicable, for the original study on which the present article is based. ***Funding is from the European Union's Seventh Framework Program (223468) and the National Institute of Nursing Research, National Institutes of Health (R01NR014855). The funders had no role in the research or the writing of this manuscript.***

*Give information separately for exposed and unexposed groups.

Note: An Explanation and Elaboration article discusses each checklist item and gives methodological background and published examples of transparent reporting. The STROBE checklist is best used in conjunction with this article (freely available on the Web sites of PLoS Medicine at http://www.plosmedicine.org/, Annals of Internal Medicine at http://www.annals.org/, and Epidemiology at http://www.epidem.com/). Information on the STROBE Initiative is available at www.strobe-statement.org.

For peer review only

BMJ Open

Patient Satisfaction with Hospital Care and Nurses in England: An Observational Study

Journal:	*BMJ Open*
Manuscript ID	bmjopen-2017-019189.R1
Article Type:	Research
Date Submitted by the Author:	07-Sep-2017
Complete List of Authors:	Aiken, Linda; University of Pennsylvania, School of Nursing<br>Sloane, Douglas; University of Pennsylvania, School of Nursing<br>Ball, Jane; National Institute for Health Research Collaboration for Leadership in Applied Health Research and Care (Wessex),University of Southampton, Centre for Innovation and Leadership in Health Sciences<br>Bruyneel, Luk; KU Leuven, Institute for Healthcare Policy<br>Rafferty, Anne Marie; King's College London, Florence Nightingale School of Nursing and Midwifery<br>Griffiths, Peter; University of Southampton, Faculty of Health Sciences
**Primary Subject Heading**:	Nursing
Secondary Subject Heading:	Health services research, Patient-centred medicine
Keywords:	patient satisfaction, hospitals, nurses, England, quality of health care

SCHOLARONE™
Manuscripts

TITLE PAGE

Patient Satisfaction with Hospital Care and Nurses in England: An Observational Study

Linda H Aiken (1)
Douglas M Sloane (1)
Jane Ball (2)
Luke Bruyneel (3)
Anne Marie Rafferty (4)
Peter Griffiths (2)

Correspondence to Linda H Aiken, Center for Health Outcomes and Policy Research, School of Nursing, University of Pennsylvania 418 Curie Boulevard, Philadelpha, PA, 19104 USA laiken@nursing.upenn.edu

Author affiliations

(1) University of Pennsylvania School of Nursing, Center for Health Outcomes and Policy Research, Philadelphia, Pennsylvania, USA
(2) Faculty of Health Sciences, University of Southampton, Southampton, England
(3) University of Leuven, Leuven Institute for Healthcare Policy, Leuven, Belgium
(4) King's College London, Florence Nightingale School of Nursing and Midwifery, London, England

Twitter: Follow Linda Aiken at @LindaAiken_Penn; Jane Ball at @JaneEBall; Luk Bruyneel at @LukBruyneel; Peter Griffiths at @workforcesoton

Acknowledgements: We thank Tim Cheney for analytic assistance, the patients, nurses, and hospitals that participated in our study, and the RN4CAST Consortium.

Contributors: LHA raised the funding, developed the idea for the study, interpreted results, drafted and revised manuscript; DMS conducted the analysis, drafted the results, contributed to manuscript revisions; JB collected survey data, contributed to manuscript development; LB contributed to results interpretation and manuscript drafting and revisions; AMR contributed to data collection, interpretation of results, manuscript revisions; PG contributed to obtaining data, results interpretation, manuscript development and revisions.

Funding: European Union's Seventh Framework Program (223468) and the National Institute of Nursing Research, National Institutes of Health (R01 NR014855).

Competing interests: None declared

Ethics approval: The study was approved by the University of Pennsylvania human subjects committee.

Keywords: Hospitals, Quality of Health Care, Nurses, England, Patient Satisfaction

Word Count: 3652

Data Sharing: No additional data are available.

For peer review only - http://bmjopen.bmj.com/site/about/guidelines.xhtml

Patient Satisfaction with Hospital Care and Nurses in England: An Observational Study

ABSTRACT (285 words)

Objectives –To inform healthcare workforce policy decisions by showing how patient perceptions of hospital care are associated with confidence in nurses and doctors, nurse staffing levels, and hospital work environments.

Design – Cross-sectional surveys of 66,348 hospital patients and 2,963 inpatient nurses.

Setting – Patients surveyed were discharged in 2010 from 161 National Health Service (NHS) trusts in England. Inpatient nurses were surveyed in 2010 in a sample of 46 hospitals in 31 of the same 161 trusts.

Participants - The 2010 NHS Survey of Inpatients obtained information from 50% of all patients discharged between June and August. The 2010 RN4CAST England Nurse Survey gathered information from inpatient medical and surgical nurses.

Main outcome measures – Patient ratings of their hospital care, their confidence in nurses and doctors , and other indicators of their satisfaction. Missed nursing care was treated as both an outcome measure and explanatory factor.

Results – Patients' perceptions of care are significantly eroded by lack of confidence in either nurses or doctors, and by increases in missed nursing care. The average number of types of missed care was negatively related to six of the eight outcomes-- odds ratios ranged from 0.78 (95% CI = 0.68 to 0.90) for excellent care ratings to 0.86 (95% CI = 0.77 to 0.95) for medications completely explained-- positively associated with higher patient-to-nurse ratios (b = 0.15, 95% CI = 0.10 to 0.19), and negatively associated with better work environments (b = -0.26, 95% CI = -0.48 to -0.04).

Conclusions – Patients' perceptions of hospital care are strongly associated with missed nursing care, which in turn is related to poor RN staffing and poor hospital work environments. Improving RN staffing in NHS hospitals holds promise for enhancing patient satisfaction.

For peer review only - http://bmjopen.bmj.com/site/about/guidelines.xhtml

Strengths and limitations of this study

- This is the first quantitative study to determine the association between patients' confidence in nurses and doctors, RN staffing, and patient experiences with hospital care in NHS hospitals in England using the national NHS Adult Inpatient Survey.

- Unique data previously unavailable enable a rigorous analysis of patient to RN staffing ratios, missed nursing care, and patient satisfaction with hospital care.

- The study uses cross-sectional data, and while a number of alternative explanations are considered in our models, we cannot rule out the possibility that omitted variables contribute to associations found.

For peer review only

For peer review only - http://bmjopen.bmj.com/site/about/guidelines.xhtml

Patient Satisfaction with Hospital Care and Nurses in England: An Observational Study

INTRODUCTION

Highly publicized reports citing preventable deaths and deficiencies in hospital care in England have uniformly concluded that inadequate hospital nurse (RN) staffing is a contributing factor (1-3). Studies confirm large variation in patient to RN ratios across National Health Service (NHS) hospitals, and this variation is associated with higher mortality in hospitals where RNs care for more patients each (4-6). However, despite national guidance on safe nurse staffing (7), substantial variation still exists and the value of higher RN staffing levels is still questioned at the policy level (8). Recently introduced NHS workforce initiatives have been framed in the unsubtantiated narrative that quality deficiences in hospitals are due to “uncaring” nurses (9,10). The National Advisory Group on the Safety of Patients in England specifically advised that nurses and other NHS staff not be blamed for quality deficits, pointing instead to the need to address insufficient RN staffing (3). Nevertheless new workforce initiatives have been introduced by the NHS purportedly to produce more caring nurses. One such initiative creates a new provider category, the nursing associate, with substantially lower qualifications than RNs (11). Adding lesser trained providers to the hospital workforce without adding more RNs results in eroding the nursing skill mix that evidence suggests is associated with higher mortality and lower patient satisfaction (12). Also, the NHS is reinstating apprendice training for RNs (13), in direct opposition to a major recommendation of the 2010 Prime Minister’s Commission on the Future of Nursing and Midwifery in England (14) that all nursing education should take place in universities because evidence shows that hospitals with a higher proportion of bachelors-prepared nurses have signficiantly better patient outcomes (6,15,16).

For peer review only - http://bmjopen.bmj.com/site/about/guidelines.xhtml

The concern about nurses being uncaring or lacking in compassion, and subsequent NHS nursing initiatives, have come about largely in response to case studies of poor care in a relatively small number of NHS trusts and anecedoctal reports of patient dissatisfaction. Surprisingly little use has been made of the NHS National Inpatient Survey of patients to inform strategies to improve care (17). When initiated in 2001, England's annual national survey of patients following a hospital inpatient stay was the first in the world; it aimed to make the NHS more patient-centered and more responsive to patient feedback (18). A report published in 2007 using the NHS Inpatient Survey found evidence that the experiences of staff working in the NHS mirrored the experiences of patients receiving care (19). This is a worrisome finding given the evidence showing high nurse burnout and job dissatisfaction is common in NHS hospitals (10, 20), and that 85% of RNs in NHS hospitals report not being able to complete needed nursing care due to lack of time associated with high patient to nurse workloads (21). Further, missed nursing care associated with high patient to nurse workloads is associated with an increased risk of patient mortality following common surgical procedures in nine European countries including England (22).

Studies of patients' experiences with inpatient care in the U.S., another country with mandated hospital patient satisfaction surveys, reveal that better RN staffing is associated with higher overall patient ratings of their hospitals (23, 24). Missed nursing care is associated with less favorable patient satisfaction in U.S. hospitals (25) and in some European hospitals (not including England) (26). There are not comparable studies in England using the NHS National Inpatient Survey that could help determine whether better RN staffing and better clinical hospital work environments are associated with more favorable patient experience with hospital care.

For peer review only

This paper seeks to identify an action agenda that may hold promise for improving patients' experiences with hospital care in England. Specifically, we first provide evidence of the importance of RNs to patients using data from a large sample of patients in NHS hospitals in England to show how patients' experience with care is strongly related to their confidence in nurses as well as doctors, and their perceptions of whether there were enough nurses in their hospitals. We then use data from patients and nurses in a subset of these hospitals to show how lower nurse workloads and better nurse work environments are related to less missed nursing care and how, in turn, less missed nursing care is related to better patients' experience with their care.

METHODS

Data sources and samples

Patient survey data are from the 2010 NHS Survey of Inpatients, which gathered information from over 66,000 patients who were discharged from 161 acute and specialist NHS trusts in England (27). Nurse survey data are from the 2010 RN4CAST-England study (28, 20) which gathered information from 2,963 inpatient medical and surgical direct care RNs in a representative sample of 31 of the same 161 NHS trusts. These 31 trusts comprise 46 different hospitals from which 12,581 of the 66,348 patients surveyed were discharged. Of these 12,851 patients, 5,311 were in general medicine or general surgery wards. The sample of hospitals in which nurses were surveyed, described elsewhere in detail (21, 28), was a stratified random sample selected to include teaching and non-teaching hospitals of different sizes in every geographic region of England. There are no remarkable differences between the sample of hospitals in which nurses were surveyed and the other hospitals participating in the NHS Survey

For peer review only - http://bmjopen.bmj.com/site/about/guidelines.xhtml

of Inpatients, nor were there any differences in patient characteristics or responses between the full NHS survey and the 31 trusts studied, as noted in the Appendix table. The response rate for the NHS patient survey was 50%. The response rate for the nurse survey was 37%. The nurse survey has good established predictive validity in previous research (6, 29), showing, for example, that nurses' reports of quality of care are closely associated with patient mortality derived from independent data sources (30).

Patients were not participants in the initial design of the overall study but were actively engaged in the development of measures of patients' experiences with care used in the study. The Picker Institute, developers of the NHS Adult Inpatient Survey, employed patient focus groups and cognitive interviews with patients during pilot testing. Patients were offered one page to describe what they thought of the inpatient questionnaire and which aspects of patient care were most important to them. The qualitative research did not identify major questions missing from the survey but it did lead to minor modifications that were incorporated (31). Patients in our study are anonymous. We have a detailed plan to disseminate the study results through print, broadcast, and social media in every participating country. We gratefully acknowledge the contributions of participating patients in the acknowledgement section.

Analysis strategy

These data were used to undertake three distinct but related analyses. First, we use patient data from all 161 trusts to describe how patients rated their care, how their ratings varied depending on their perceptions of whether there were enough nurses on duty to provide needed care, and how they were as much a function of their confidence in nurses as their confidence in doctors. We then used the nurse data from the 46 hospitals in the 31 trusts to describe the

For peer review only - http://bmjopen.bmj.com/site/about/guidelines.xhtml

variation in RN staffing and hospital work environments, and then used least-squares regression models with and without control variables to show how lower RN staffing levels and poorer work environments are related to needed but missed nursing care. Finally, since patient survey data were only available at the trust level, we merged the nurse data from the 31 trusts with patient data from those same trusts and used logistic regression models to estimate whether and to what extent the overall level of missed nursing care in the different trusts affect patients' ratings of their care and their confidence in nurses, before and after controlling for potential confounds. Because the nurse survey was restricted to nurses on medical and surgical units, this final step of the analysis was restricted to patients in general surgical and medical wards (5,311 out of 12,851 patients in the study trusts).

RESULTS

Nurses, Doctors, and Patient Ratings of Care.

Table 1 and **Figure 1** use data from 66,348 patients in 161 trusts collected in the 2010 NHS Survey of Inpatients to show how patients' ratings of their care are highly associated with their confidence in nurses and in doctors, and with their perceptions of whether there were enough nurses to provide needed care. The first column of Table 1 shows that more than three fourths of patients responding to the NHS survey reported having confidence and trust in the doctors and nurses treating them, while only 60 percent reported that there were always or nearly always enough nurses to care for them. The second column of Table 1 shows the percentages of patients that rated their care as excellent, based on how much confidence and trust they had in their nurses and doctors, and their perceptions of the adequacy of the number of nurses caring for them. Hospital care was rated excellent by over half of the patients who indicated that they always had confidence and trust in their doctors or confidence and trust in their nurses, but by

For peer review only - http://bmjopen.bmj.com/site/about/guidelines.xhtml

only 3% of the patients who never had confidence and trust in their doctors or in their nurses. Similarly, hospital care was rated as excellent by over half of the patients who indicated that there were always enough nurses to care for them, but by far lower percentages of patients who said there were only sometimes enough, or rarely or never enough, nurses.

While Table 1 makes it clear that nurses, like doctors, are importantly related to patients' perceptions of the quality of their care, Figure 1 shows more directly that confidence and trust in nurses is of similar importance to confidence and trust in doctors. In Figure 1 we show the percent of patients that rated their care "excellent" after grouping the patients into four categories, to distinguish patients who always have confidence and trust in both doctors and nurses, in doctors but not nurses, nurses but not doctors, and neither doctors not nurses. Sixty percent of patients who have confidence and trust in both doctors and nurses rate their care as excellent, while only three percent of patients who have confidence and trust in neither rate their care as excellent. When confidence and trust in either group erodes, the result is virtually identical. Only 16% of the patients who have confidence and trust in their doctors but not nurses rate their care as excellent, and only 17% of the patients who have confidence and trust in their nurses but not doctors rate their care as excellent.

For peer review only

Table 1. Patient Reports about Nurses and Doctors, and the Percent Indicating Their Care was "Excellent," Based on Their Reports about Doctors and Nurses.

Patient Survey Question		Percent of Patients in Each Response Category	Percent of Patients in Each Response Category Indicating That Their Care was "Excellent"
Did you have confidence and trust in the doctors treating you?	Yes, always	80.4%	52.6%
	Yes, sometimes	16.4%	9.4%
	No	3.1%	3.4%
	Total	100.0%	
Did you have confidence and trust in the nurses treating you?	Yes, always	75.1%	55.3%
	Yes, sometimes	21.7%	10.5%
	No	3.2%	2.8%
	Total	100.0%	
Were there enough nurses on duty to care for you in the hospital?	Always or nearly always	60.4%	57.3%
	Sometimes	29.5%	26.7%
	Never or rarely	10.1%	14.1%
	Total	100.0%	

Source: Data are from the 2010 NHS Survey of Inpatients, which involved 66,348patients discharged from 161 trusts in England.

Note: The numbers reported exclude a small number (< 2%) of missing responses.

Nurse Staffing, Work Environments, and Missed Nursing Care

Nurse (RN) staffing was estimated for the 46 hospitals included in RN4CAST-England by average nurse workloads in each hospital on the day shift. Nurses reported how many patients they cared for on their last shift, and then responses are averaged across all nurses in each hospital working the day shift. Nurse workloads averaged 8.6 patients per RN during the day, and ranged from 5.6 patients per RN to 11.5 patients per RN across the 46 hospitals. Patient to

For peer review only - http://bmjopen.bmj.com/site/about/guidelines.xhtml

RN ratios were much higher and more variable at night so we elected to use day shift staffing only in our analyses.

Hospital work environment was measured by the Practice Environment Scale of the Nursing Work Index (PES-NWI), an extensively used survey-based measure with established reliability and validity (32-35) leading to its adoption by the National Quality Forum as a nurse sensitive quality of care indicator (36). The measure of work environment used is a composite measure formed from 5 subscales (comprised of 28 nurse survey items) measuring resource adequacy (4 items), nurse participation in hospital affairs (8 items), nursing foundations for quality care (9 items), nurse manager ability, leadership, and support of nurses (4 items), and nurse-physician relations (3 items). The staffing and resource adequacy subscale was dropped from the global measure used in the analysis because of its high correlation with the direct measure of RN staffing in the model, as in previous publications (37, 12).

What makes the variability in staffing and work environments across hospitals of considerable importance is that when RNs have high patient loads, and when RNs practice in poor work environments, necessary nursing care can be missed because of lack of time (21). Nurses in this study were asked whether any of thirteen important types of nursing care were needed but missed because of lack of time. **Figure 2** shows that while 7% of nurses reported that they lacked time to complete necessary pain management, and 11% missed treatments and procedures, much greater percentages reported lacking the time to educate patients and their families (52%) and comfort or talk with their patients (65%). More than a quarter of the nurses (27%) lacked the time to complete three or four of the types of care listed, just under one in five (19%) lacked the time to complete five or six of them, and another 19% lacked the time to complete seven or more of the 13 types of care listed.

For peer review only - http://bmjopen.bmj.com/site/about/guidelines.xhtml

For peer review only

Table 2 provides regression coefficients that indicate the effects of RN staffing and the hospital work environment on the average number of types of missed care, before and after controlling for various hospital characteristics (including size, technology, and location), and characteristics of nurses that may have affected their reports of missed care, including their role (primary nurse or shared responsibility for group of patients with other nurses), full-time status, years of experience, and unit type (medical, surgical, or combined). Higher nurse workloads (higher patient to RN ratios) are significantly related to higher numbers of types of missed care, while better work environments are significantly related to fewer types of missed care, both before and after adjustment.

Table 2. Regression Coefficients Indicating the Effects of Staffing and Practice Environment on Average Number of Types of Care Missed

	**Regression Coefficients [and 95% Confidence Intervals]**	
**Effect on Missed Care of -**	**Unadjusted**	**Adjusted**
**Patient to Nurse Ratio**	0.11***	0.15***
	[0.06,0.16]	[0.10,0.19]
**Practice Environment**	-0.30*	-0.26*
	[-0.55,-0.05]	[-0.48,-0.04]

Source: Data are from the 2010 RN4CAST-England study, which surveyed 2,963 inpatient medical and surgical direct care professional nurses (RNs) in a representative sample of 31 NHS trusts comprised of 46 different hospitals.

Notes: Adjusted Coefficients and confidence intervals are from regression models which control for hospital characteristics (Beds > 750, High Technology, and Location) and nurse characteristics (Nurse Role, Full-time Status, Years of Experience and Unit Type). Practice environment is measured by the PES-NWI tertile.

Single, double and triple asterisks denote coefficients that are significant at the .05, .01, and .001 levels, respectively.

Figures 3 and 4 show how much the number of tasks left undone varies as a function of RN staffing and hospital work environments, as estimated from the adjusted models. As the

For peer review only - http://bmjopen.bmj.com/site/about/guidelines.xhtml

number of patients per RN goes down, from 12 patients to 8 patients to 4 patients, the average number of types of missed care goes down, from 4.4 (out of 13) to 3.8 to 3.2. And, as hospital work environments improve, from relatively poor (lowest tertile) to average (middle tertile) to relatively good (highest tertile), the average number of types of missed care also goes down, from 4.2 to 4.0 to 3.7.

Missed Nursing Care and Patient Outcomes

The association of the number of types of missed care with patient outcomes is shown in **Table 3**. The coefficients in the table are odds ratios which indicate how much the odds on providing a positive response to the nine different dimensions of patient satisfaction go down as the average number of types of missed care goes up, both before (unadjusted) and after (adjusted) taking account patient characteristics that might affect their responses, including gender, age, length of stay, ward, number of long standing conditions, and type of admission (emergent/urgent or planned). In all cases, the odds ratios are less than one, indicating that positive patient appraisals of care decrease as the number of types of missed care increases; in six of the eight aspects of patient care rated the odds ratios are significant, and range from 0.78 to 0.86. These values indicate, for example, that in hospitals in which the number of types of missed care averaged 4.5 per nurse per shift, the odds on patients rating care as excellent and responding that the purpose of medicines were completely explained were 22% lower and 14% lower, respectively, than in hospitals in which the number of types of missed care averaged 3.5 per nurse per shift.

For peer review only - http://bmjopen.bmj.com/site/about/guidelines.xhtml

Table 3. Odds Ratios Indicating the Effect of the Trust Median Number of Types of Care Missed on Various Patient Outcomes

Effect of the Median Number of Types of Care Missed on Patient Outcomes	Odds Ratios [and 95% Confidence Intervals] Unadjusted	Adjusted
Rate Care Excellent	0.79*** [0.69,0.90]	0.78*** [0.68,0.90]
Did Not Want To Complain About Care	0.92 [0.76,1.12]	0.92 [0.77,1.11]
Always Felt Treated with Respect and Dignity	0.89 [0.78,1.02]	0.92 [0.81,1.06]
Completely Explained Purpose of Medicines	0.87* [0.78,0.98]	0.86** [0.77,0.95]
Doctors and Nurses Work Together Excellent	0.84** [0.74,0.94]	0.82** [0.72,0.93]
Always Got Answers I Could Understand	0.84** [0.75,0.95]	0.83*** [0.76,0.91]
Always Have Confidence and Trust In Nurses	0.86* [0.74,0.99]	0.85* [0.73,0.99]
Always or Nearly Always Enough Nurses	0.87* [0.76,0.99]	0.85** [0.75,0.96]

Source: Data are from a merged file that included information from 31 NHS trusts for which both patient information (from 5,311 general medical and surgical patients included in the 2010 NHS Survey of Inpatients) and nurse information (from 2,963 medical and surgical nurses surveyed in the 2010 RN4CAST-England study) were available.

Notes: Adjusted models control for hospital characteristics(Beds > 750, High Technology, and Location) and patient characteristics that might affect responses, including gender, age, length of stay, ward, number of long standing conditions and type of admission (emergent/urgent or planned).

Single, double and triple asterisks denote coefficients that are significant at the .05, .01, and .001 levels, respectively.

For peer review only - http://bmjopen.bmj.com/site/about/guidelines.xhtml

DISCUSSION

National survey data from patients experiencing a hospitalization in an NHS hospital in England confirm that patients have a high level of trust and confidence in RNs, evidence that refutes the narrative blaming quality of care deficits in NHS hospitals on uncaring nurses. However, only 60 percent of patients indicated that there were always enough RNs to care for them, and one in 10 patients indicated that there were never or rarely enough RNs. The importance to patients of adequate RN staffing is evident in their responses; 57% of patients who indicated that there were always or nearly always enough RNs to care for them rated care as excellent, compared with only 14% of the patients who said there were rarely or never enough. Additional analyses undertaken (not shown) indicate that patients in hospitals with poorer RN staffing are much less likely to say there were always enough nurses to care for them. We estimate, from models that took account of numerous confounds, that the likelihood of patients saying there were always enough nurses to take care of them were about 40% lower in hospitals in which the average nurse took care of 10 patients than in hospitals in which the average nurse took care of 6 patients. These findings reinforce from patients' perspectives the importance of adequate hospital RN staffing.

Further insights into how quality of care might be improved in NHS hospitals is revealed when data from the NHS Inpatient Survey is linked with information on actual hospital RN staffing and nurses' assessments of the quality of their work environments. We found substantial variation across NHS general acute hospitals in patient-to-nurse workloads. Nurses in some NHS hospitals are caring for twice as many patients at a time as nurses in other hospitals. Current NHS policies devolving greater autonomy to hospital management to make decisions about RN staffing may be contributing to the substantial observed variation in staffing, and have led

For peer review only - http://bmjopen.bmj.com/site/about/guidelines.xhtml

experts to point to the need for checks and balances to minimize the risk of more quality failures linked to inadequate RN staffing (38). Our findings show that the substantial differences in RN staffing across NHS hospitals are associated with the extent to which needed nursing care is missed. The most frequently missed types of care include those that patients may readily recognize are missing--comforting and talking with patients, and teaching patients and family members how to manage care following discharge. Our results are consistent with other research showing that higher patient workloads for RNs in NHS hospitals are associated with adverse patient outcomes including higher hospital morality (4, 5, 22). Initiatives such as those recently adopted in Wales (39) establishing an upper limit to how many patients nurses can safely and effectively care for holds promise for further improvements in patients' satisfaction with hospital care, and may save lives as well.

Another modifiable feature of hospital care found to be relevant to patients' perceptions of their care is the quality of the hospital work environment. In hospitals rated by nurses to have less favorable clinical work environments, needed but missed nursing care is more extensive. Patients' perceptions of care are less favorable when missed care is more extensive. Research suggests that hospital work environments that support RNs to provide care efficiently and effectively, and without constant interruptions because of operational failures such as missing medications and equipment (40), are reasonably low cost interventions and return good value in terms of better patient outcomes at the same or lower costs (41,42). Magnet hospitals formally recognized for their good hospital work environments have significantly higher patient satisfaction than matched non-Magnet hospitals (43). One of the first Magnet hospitals accredited outside the U.S. was a NHS trust in England, which research showed significantly improved its work environment and care quality during the process of achieving Magnet

For peer review only - http://bmjopen.bmj.com/site/about/guidelines.xhtml

accreditation (44). Unfortunately, the NHS merged the Magnet facility out of existence after a year, and there has not been a Magnet accredited hospital in England in over 15 years.

Patients' confidence in both doctors and nurses is equally important in how patients rate their hospitals; few patients that have high confidence in their doctors but little confidence in their nurses rate their hospitals highly. This finding is relevant to policy decisions governing the composition of the NHS workforce in England. Between 2010 and 2015, the number of physician consultants (mostly inpatient physicians) increased by more than one-fifth while the number of RNs increased only one percent (45).

Our study has many strengths including use of validated measures of patient satisfaction, nurse staffing, hospital work environment, and missed nursing care across large numbers of NHS hospitals. The study has limitations as well. Data from both patients and nurses is cross sectional thus limiting causal inferences about the associations found. We take into account, to the extent possible, alternative explanations about factors that could be associated with our findings including characteristics of hospitals such as teaching status, and characteristics of the patients responding to the national survey, including their health status since self-reported limiting long-term conditions have been found to be associated with less favorable perceptions of care (46). Our data are from 2010 but remain the only comprehensive data on hospital nurse workforce and patient satisfaction across large numbers of NHS hospitals in England. Moreover, our interest is in the relationship between patient satisfaction and nurse resources, and there is no reason to expect the relationship to have changed since 2010. Indeed, Sir Robert Francis, author of the public inquiry into quality of care deficiencies at the Mid Staffordshire NHS Trust (1), commented as recently as July 2017 that safe nurse staffing in England still lacks a standardized approach and substantial variation across hospitals in nurse staffing remains (47).

For peer review only - http://bmjopen.bmj.com/site/about/guidelines.xhtml

CONCLUSIONS

Patients express a high level of confidence and trust in nurses, and their satisfaction with hospital care is less favorable when they perceive there are not enough nurses available. The narrative that quality deficits in hospitals in England are due to “uncaring” nurses is not supported by the evidence. To the contrary, our findings suggest that reducing missed nursing care by ensuring adequate numbers of RNs at the hospital bedside and improved hospital clinical care environments are promising strategies for enhancing patient satisfaction with care.

For peer review only

For peer review only - http://bmjopen.bmj.com/site/about/guidelines.xhtml

Acknowledgements: We thank Tim Cheney for analytic assistance, the patients, nurses, and hospitals that participated in our study, and the RN4CAST Consortium.

Competing interests: None declared

Funding: European Union's Seventh Framework Program (223468) and the National Institute of Nursing Research, National Institutes of Health (R01NR014855).

For peer review only

For peer review only - http://bmjopen.bmj.com/site/about/guidelines.xhtml

REFERENCES

1. Francis R. Report of the Mid Staffordshire NHS Foundation Trust Public Inquiry; 2013. Available from: http://webarchive.nationalarchives.gov.uk/20150407084003/http://www.midstaffspublicinquiry.com/report.
2. Keogh B. Review into the quality of care and treatment provided by 14 hospital trusts in England: overview report; 2013. Available from: http://www.nhs.uk/NHSEngland/bruce-keogh-review/Documents/outcomes/keogh-review-final-report.pdf.
3. National Advisory Board on the Safety of Patients in England. A promise to learn–a commitment to act: improving the safety of patients in England. London: Department of Health; 2013. Available from: www.health.org.uk/government/publications/berwick-review.
4. Rafferty AM, Clarke SP, Coles J, Ball J, James P, McKee M, et al. Outcomes of variation in hospital nurse staffing in English hospitals: cross-sectional analysis of survey data and discharge records. Int J Nurs Stud. 2007;44(2):175-82.
5. Griffiths P, Ball J, Murrells T, Jones S, Rafferty AM. Registered nurse, healthcare support worker, medical staffing levels and mortality in English hospital trusts: a cross-sectional study. BMJ Open. 2016;6(2):e008751.
6. Aiken LH, Sloane DM, Bruyneel L, Van den Heede K, Griffiths P, Busse R, et al. Nurse staffing and education and hospital mortality in nine European countries: a retrospective observational study. Lancet. 2114;383;1824-30.
7. National Institute for Health and Care Excellence (NICE). NHS England asks NICE to suspend safe staffing programme. United Kingdom: NICE, 2015. Available from: https://www.nice.org.uk/news/article/nhs-england-asks-nice-to-suspend-safe-staffing-programme.
8. Lord Carter of Coles. Operational productivity and performance in English NHS acute hospitals: unwarranted variation. February 2016. Available from: https://www.gov.uk/government/uploads/system/uploads/attachment_data/file/498107/Carter_Review_-_executive_summary.pdf.
9. Smith R. Nurses lack compassion NHS admits. The Telegraph. 2012 January 18.
10. Aiken L, Rafferty AM, Sermeus W. Caring nurses hit by a quality storm. Nursing Standard. 2014;28(35):22-5.
11. Gummer B. Nursing associate role offers new route into nursing. UK: Department of Health. [cited 2016 Jan 25]. Available from: http://www.gov.uk/government/news/nursing-associate-role-offers-new-route-into-nursing.
12. Aiken LH, Sloane D, Griffiths P, Rafferty AM, Bruyneel L, McHugh M, et al. Nursing skill mix in European hospitals: cross-sectional study of the association with mortality, patient ratings, and quality of care. BMJ Quality & Safety. 2016. Epub 2016 Nov 15.
13. Department of Health and Human Services. New nursing degree apprenticeship United Kingdon: GOV.UK; 2016. Available from: https://www.gov.uk/government/news/new-nursing-degree-apprenticeship.
14. Prime Minister's Commission on the Future of Nursing and Midwifery in England. Front Line. 2010. Available from: http://webarchives.gov.uk/20100331110913.
15. Aiken LH, Clarke SP, Cheung RB, Sloane DM, Silber JH. Educational levels of hospital nurses and surgical patient mortality. JAMA. 2003; 290(12);1617-1623.
16. Yakusheva O, Lindrooth R, Weiss M. Economic evaluation of the 80% baccalaureate nurse workforce recommendation.: a patient level analysis. Medical Care. 2014;52;864-869.

For peer review only - http://bmjopen.bmj.com/site/about/guidelines.xhtml

17. Coulter A, Locock L, Ziebland S, Calabrese J. Collecting data on patient experience is not enough: they must be used to improve care. BMJ. 2014;348:g2225.
18. DeCourcy A, West E, Barron D. The National Adult Inpatient Survey conducted in the English National Health Service from 2002 to 2009: how have the data been used and what do we know as a result? BMC Health Services Research. 2012;12:71.
19. Dawson J. Does the experience of staff working in the NHS link to the patient experience of care? An analysis of links between the 2007 acute trust inpatient and NHS staff surveys. Institute for Health Services Effectiveness. 2009 [cited 2017 Jun 9]. Available from: https://www.gov.uk/government/uploads/system/uploads/attachment_data/file/215457/dh_129662.pdf.
20. Aiken LH, Sloane DM, Bruyneel L, Van den Heede K, Sermeus W. Nurses' reports of working conditions and hospital quality of care in 12 countries in Europe. Int J Nurs Stud. 2013;50(2):143-53.
21. Ball JE, Murrells T, Rafferty AM, Morrow E, Griffiths P. 'Care left undone' during nursing shifts: associations with workload and perceived quality of care. BMJ Quality & Safety. 2014 Feb;23(2):116-25.
22. Ball J, Bruyneel L, Aiken LH, Sermeus W, Sloane DM, Rafferty AM, Lindqvist R, Tishelman C, Griffiths P. Post-operative mortality, missed care and nurse staffing in nine countries: a cross sectional study. Int J Nurs Studies. In press 2017.
23. Jha AK, Orav EJ, Zheng J, Epstein AM. Patients' perception of hospital care in the United States. N Engl J Med. 2008;359(18):1921-31.
24. Kutney-Lee A, McHugh MD, Sloane DM, Cimiotti JP, Flynn L, Neff DF, Aiken LH. Nursing: A key to patient satisfaction. Heath Affairs. 2009; 28(4);w669-677.
25. Lake ET, Germack HD, Viscardi MR. Missed nursing care is linked to patient satisfaction: a cross sectional study of U.S. hospitals. BMJ Quality and Safety. 2016;25:535-543.
26. Bruyneel L, Sloane DM, Smith HL, Lesaffre E, Sermeus W, Aiken LH. Organization of hospital nursing, provision of nursing care, and patient assessments of care in Europe. Medical Care Research and Review. 2015;72(6):643-664.
27. Care Quality Commission. National NHS patient survey programme Survey of adult inpatients 2010 England: NHS; 2011. Available from: http://www.nhssurveys.org/survey/1017.
28. Sermeus W, Aiken LH, Van den Heede K, Rafferty AM, Griffiths P, Moreno-Casbas MT, et al. Nurse forecasting in Europe (RN4CAST): rationale, design and methodology. BMC Nurs. 2011;10(1):6.
29. Aiken LH, Sermeus W, Van den Heede K, Sloane DM et al. Patient safety, satisfaction, and quality of care: cross-sectional surveys of nurses and patients in 12 countries in Europe and the United States. BMJ. 2012;344;e1717.
30. Smeds-Alenius L, Tishelman C, Lindqvist R, Runesdotter S, McHugh MD. RN assessments of excellent quality of care and patient safety are associated with significantly lower odds of 30-day inpatient mortality: A national cross-sectional study of acute-care hospitals. Int J Nurs Stud. 2016;61:117-124.
31. Reeves R, Coulter A, Jenkinson C, Cartwright J, Bruster S, Richards N: Development and pilot testing of questionnaires for use in the acute NHS trust inpatient survey programme. Europe: Picker Institute [cited 2016 Jan 29]. Accessible from: http://www.nhssurveys.org/Filestore/documents/DevelopmentInpatientQuestionnaire.pdf.

For peer review only - http://bmjopen.bmj.com/site/about/guidelines.xhtml

32. Lake ET. Development of the Practice Environment Scale of the Nursing Work Index. Res Nurs Health. 2002;25(3):176-88.
33. Lake ET. The nursing practice environment: measurement and evidence. Med Care Res Rev. 2007;64(2 Suppl):104S-22S.
34. Aiken LH, Clarke SP, Sloane DM, Lake ET, Cheney T. Effects of hospital care environment on patient mortality and nurse outcomes. J Nurs Adm. 2008;38(5):223-9.
35. Swiger PA, Patrician PA Miltner RS, Raju D, et al. The Practice Environment Scale of the Nursing Work Index: updated review and recommendations for use. Int J Nurs Studies. 2017;74:76-84.
36. National Quality Forum. National Voluntary Consensus Standards for Nursing-Sensitive Care: An initial performance measure set - A consensus report. Washington D.C.; 2004. Report No.: NQFCR-08-04.
37. Aiken LH, Cimiotti J, Sloane DM, Smith HL, Flynn L, Neff D. The effects of nurse staffing and nurse education on patient deaths in hospitals with different nurse work environments. Medical Care. 2011;49(12):1047-1053.
38. Buchan J, Seccombe I, Gershlick B, Charlesworth A. In short supply: pay policy and nurse numbers. London: The Health Foundation, 2017 [updated April 2017]. Available from: http://www.health.org.uk/sites/health/files/Workforce%20pressure%20points%202017%20FINAL_0.pdf.
39. National Assembly for Wales. Nurse Staffing Levels (Wales) Act 2016, United Kingdom. 2016. Accessed from: http://legislation.data.gov.uk/anaw/2016/5/data.htm?wrap=true.
40. Tucker AL, Spear SJ. Operational failures and interruptions in hospital nursing. Health Services Research 2006;41:643-662.
41. Silber J, Rosenbaum P, McHugh M, Ludwig J, Smith H, Niknam B, et al. Comparison of the value of nursing work environments in hospitals across different levels of patient risk. JAMA Surgery. 2016;151(6):527-36.
42. Kutney-Lee A, Stimpfel AW, Sloane DM, Cimiotti JP, Quinn LW, Aiken LH. Changes in patient and nurse outcomes associated with Magnet hospital recognition. Med Care. 2015;53(6):550-7.
43. Stimpfel AW, Sloane DM, McHugh MD, Aiken LH. Hospitals known for nursing excellence associated with better hospital experiences for patients. Health Serv Res. 2015;51(3):1120-34.
44. Aiken LH, Buchan J, Ball J, Rafferty AM. Transformative impact of Magnet designation: England case sudy. J Clin Nurs. 2008;17(24);3330-3337.
45. Lafond S, Charlesworth A, Roberts A. A year of plenty? An analysis of NHS finances and consultant productivity, United Kingdom; 2017 [cited 2017 June 9]. Available from: www.health.org.uk/publication/year-of-plenty.
46. Hewitson P, Skew A, Graham C, Jenkinson C, Coulter A. People with limiting long-term conditions report poorer experiences and more problems with hospital care. BMC Health Services Research. 2014;14(1):33.44.
47. Safe staffing still lacks standardized approach, warns Francis. Nursing Times. July 6, 2017. Available from: https://www.nursingtimes.net/7019357article?utm_source=newslettertutm_medium=email&utm.

For peer review only - http://bmjopen.bmj.com/site/about/guidelines.xhtml

Figure 1. Percent of Patients Rating Their Care Excellent, by Confidence and Trust in Nurses and Doctors

Source: Data are from the 2010 NHS Survey of Inpatients, which involved 66,348 patients discharged from 161 trusts in England.

Figure 2. Percent of of Nurses Reporting That Different Types of Care Were Missed on Their Last Shift

Source: Data are from the 2010 RN4CAST-England study, which surveyed 2,963 inpatient medical and surgical direct care professional nurses (RNs) in a representative sample of 31 NHS trusts comprised of 46 different hospitals.

Figure 3. Average Number of Types of Missed Care, by Nurse Workload

Source: Data are from the 2010 RN4CAST-England study, which surveyed 2,963 inpatient medical and surgical direct care professional nurses (RNs) in a representative sample of 31 NHS trusts comprised of 46 different hospitals.

Figure 4. Average Number of Types of Missed Care, by Work Environment

Source: Data are from the 2010 RN4CAST-England study, which surveyed 2,963 inpatient medical and surgical direct care professional nurses (RNs) in a representative sample of 31 NHS trusts comprised of 46 different hospitals.

For peer review only

For peer review only - http://bmjopen.bmj.com/site/about/guidelines.xhtml

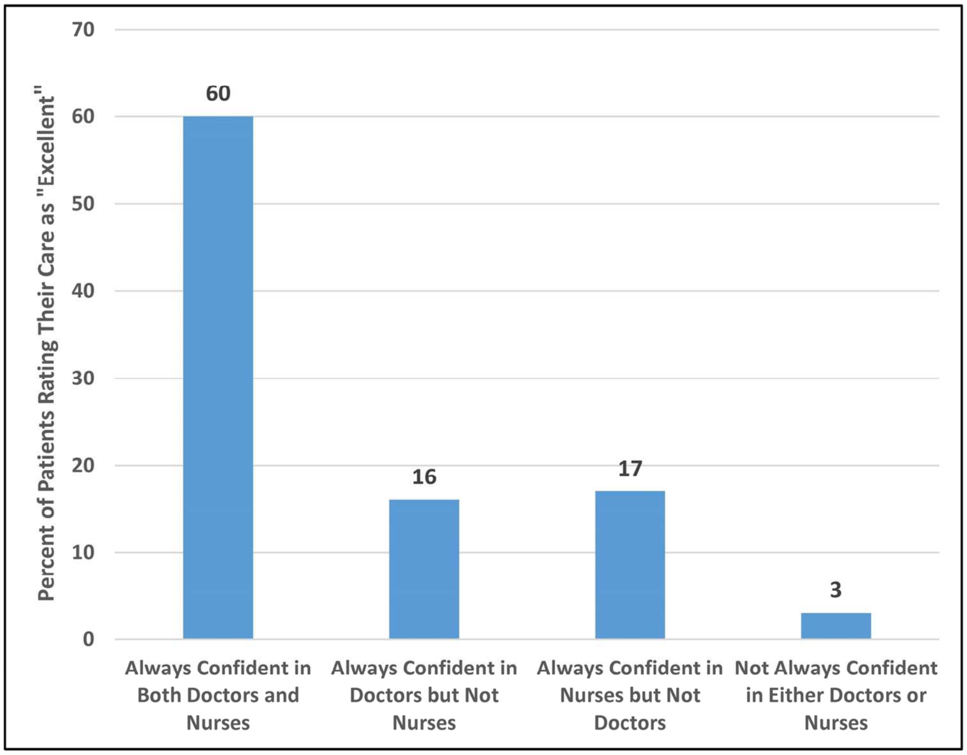

Figure 1. Percent of Patients Rating Their Care Excellent, by Confidence and Trust in Nurses and Doctors

Source: Data are from the 2010 NHS Survey of Inpatients, which involved 66,348 patients discharged from 161 trusts in England.

134x105mm (300 x 300 DPI)

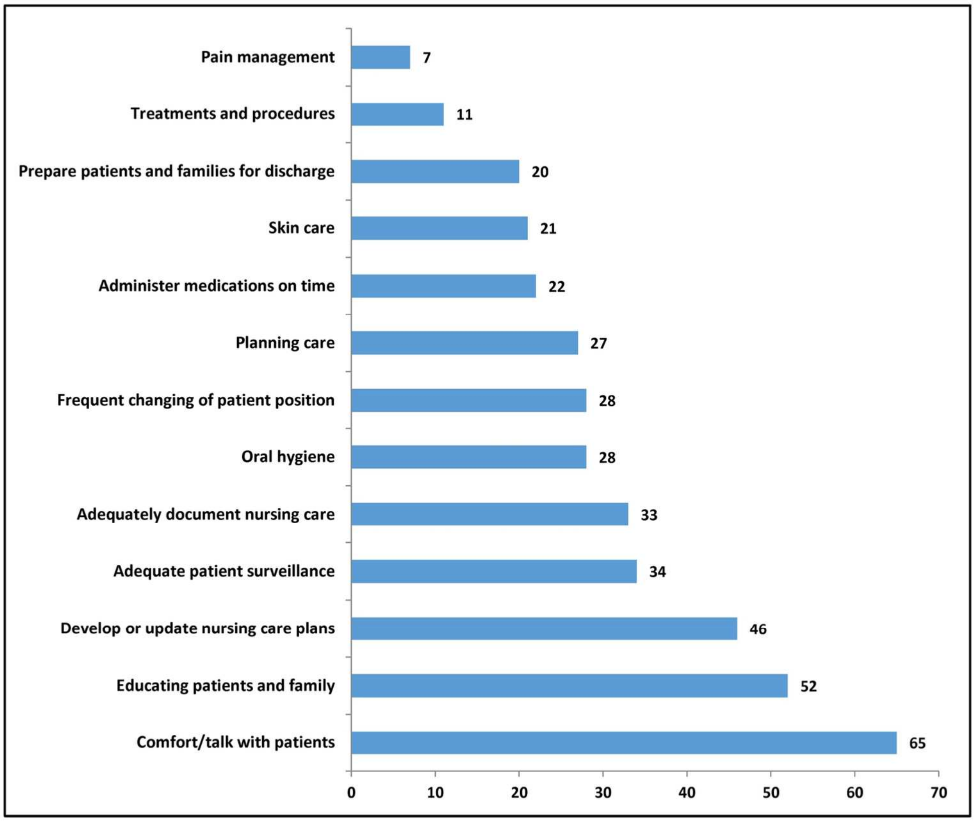

Figure 2. Percent of of Nurses Reporting That Different Types of Care Were Missed on Their Last Shift

Source: Data are from the 2010 RN4CAST-England study, which surveyed 2,963 inpatient medical and surgical direct care professional nurses (RNs) in a representative sample of 31 NHS trusts comprised of 46 different hospitals.

141x118mm (300 x 300 DPI)

nly

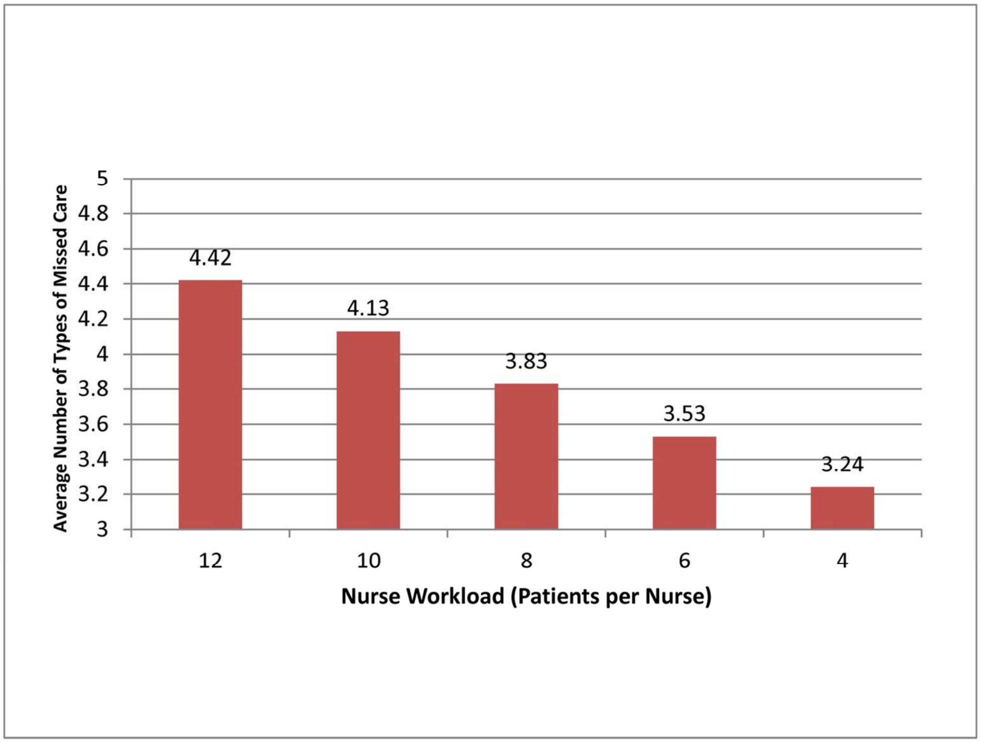

Figure 3. Average Number of Types of Missed Care, by Nurse Workload

Source: Data are from the 2010 RN4CAST-England study, which surveyed 2,963 inpatient medical and surgical direct care professional nurses (RNs) in a representative sample of 31 NHS trusts comprised of 46 different hospitals.

131x99mm (300 x 300 DPI)

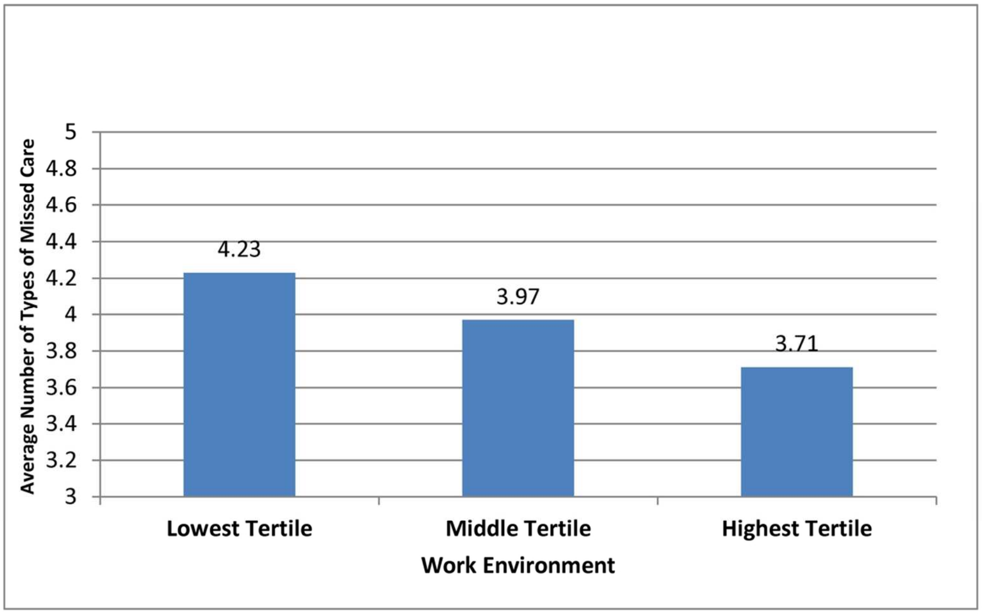

Figure 4. Average Number of Types of Missed Care, by Work Environment

Source: Data are from the 2010 RN4CAST-England study, which surveyed 2,963 inpatient medical and surgical direct care professional nurses (RNs) in a representative sample of 31 NHS trusts comprised of 46 different hospitals.

108x68mm (300 x 300 DPI)

Appendix

Patient Responses To Survey Items Related to Their Care.

Survey Question	NHS Survey Sample Number	NHS Survey Sample Percent	RN4CAST Sample Number	RN4CAST Sample Percent
**Overall Rating of Care**				
Very Good, Good, Fair or Poor	11,290	61.3	3,183	62.0
Excellent	7,128	38.7	1,950	38.0
Total	18,418	100.0	5,133	100.0
**Wanted To Complain About Your Care**				
Yes	1,727	9.5	489	9.7
No	16,422	90.5	4,554	90.3
Total	18,149	100.0	5,043	100.0
**Felt Treated with Respect and Dignity**				
No or Only Sometimes	4,400	24.3	1,254	24.3
Always	14,031	75.7	3,899	75.7
Total	18,431	100.0	5,153	100.0
**Completely Explained Purpose of Medicines***				
No or Only to Some Extent	3,909	28.7	1,170	30.4
Yes Completely	9,726	71.3	2,684	69.6
Total	13,635	100.0	3,854	100.0
**Doctors and Nurses Work Together Excellent**				
Very Good, Good, Fair or Poor	11,929	64.9	3,325	64.9
Excellent	6,443	35.1	1,801	35.1
Total	18,372	100.0	5,126	100.0
**Got Answers I Could Understand**				
No or Only Sometimes	6,330	37.4	1,790	37.9
Always	10,596	62.6	2,933	62.1
Total	16,926	100.0	4,723	100.0
**Always Have Confidence and Trust In Nurses**				
No or Only Sometimes	5,197	27.6	1,413	27.1
Always	13,619	72.4	3,806	72.9
Total	18,816	100.0	5,219	100.0
**Always Have Confidence and Trust In Doctors**				
No or Only Sometimes	4,636	24.7	1,294	24.9
Always	14,112	75.3	3,905	75.1
Total	18,748	100.0	5,199	100.0
**Enough Nurses on Duty to Care for You**				
Sometimes, Rarely or Never	8,343	44.6	2,363	45.5
Always or Nearly Always	10,378	55.4	2,832	54.5
Total	18,721	100.0	5,195	100.0

Note: Survey items for which differences are significant (at p < .05) are denoted by asterisks.

For peer review only - http://bmjopen.bmj.com/site/about/guidelines.xhtml

STROBE Statement—Checklist of items that should be included in reports of ***cross-sectional studies*** (***Note: page numbers below refer to the page numbers at the bottom of each page in the document, not the electronic page numbers).***

	Item No	Recommendation
**Title and abstract**	1	(*a*) Indicate the study's design with a commonly used term in the title or the abstract ***The observational and cross-sectional study design is noted in the Title Page (p. i) and Abstract page (p. ii, Title and Design sections).***
		(*b*) Provide in the abstract an informative and balanced summary of what was done and what was found ***The study design, settings, participants, outcome measure and results are described in the Abstract (p. ii).***
**Introduction**		
Background/rationale	2	Explain the scientific background and rationale for the investigation being reported ***The background and rationale are explained in the "Introduction" (p. 1-2)***
Objectives	3	State specific objectives, including any prespecified hypotheses ***The primary objective of the study is stated in the last paragraph of the introduction (p. 2)***
**Methods**		
Study design	4	Present key elements of study design early in the paper. ***Key elements of the study design are described in the Abstract (pg. ii) and in the Methods section of the paper (p. 3-4)***
Setting	5	Describe the setting, locations, and relevant dates, including periods of recruitment, exposure, follow-up, and data collection. ***The setting, locations and dates of the trusts, patients and nurses from which data were collected are given in the first paragraph of the Methods section (p. 3).***
Participants	6	(*a*) Give the eligibility criteria, and the sources and methods of selection of participants. ***We describe the sampling of trusts, patients and nurses in the Methods section (p. 3). We also reference prior publications which contain additional details in the text on that page.***
Variables	7	Clearly define all outcomes, exposures, predictors, potential confounders, and effect modifiers. Give diagnostic criteria, if applicable. ***Key patient variables (including patient ratings of care and satisfaction, and nurse staffing, work environments, and missed nursing car)e are described in general on p. 5-6, and additional details on control variables used are given in the note below Table 2 (p. 9).***
Data sources/ measurement	8*	For each variable of interest, give sources of data and details of methods of assessment (measurement). Describe comparability of assessment methods if there is more than one group. ***Data are from the NHS patient satisfaction survey and RN4CAST nurse survey as described on p. 3.***
Bias	9	Describe any efforts to address potential sources of bias ***We attempted to obtain unbiased results by using logistic regression models before and after adjusting for hospital, nurse and patients characteristics, as indicated in the text and notes related to Tables 2 (p. 9) and 3 (p. 11).***
Study size	10	Explain how the study size was arrived at ***We describe the sampling of trusts, patients and nurses in the Methods section (p. 3). We also reference prior publications which contain additional details in the text on p. 3.***

For peer review only - http://bmjopen.bmj.com/site/about/guidelines.xhtml

Quantitative variables	11	Explain how quantitative variables were handled in the analyses. If applicable, describe which groupings were chosen and why. ***Some quantitative variables (i.e. nurse staffing, nurse experience, number of types of missed care) were treated as continuous and not grouped. We describe grouping of work environment in the Methods section (p. 3). Numerous control variables were dichotomous and represented by dummy variables [see notes to Tables 2 (p. 9) and 3 (p. 11) and related text.***
Statistical methods	12	(*a*) Describe all statistical methods, including those used to control for confounding ***We discuss the least squares and logistic regression models we used in the Analysis Strategy sub-section of the Methods section (p. 4).***
		(*b*) Describe any methods used to examine subgroups and interactions ***We have not, as yet, investigated subgroup differences and interactions with these data.***
		(*c*) Explain how missing data were addressed ***Information on missing data for the NHS Inpatient Survey are negligible, and described in previous work that we have referenced. We not that for the measures we considered (cf., Note for Table 1, p. 7) that the numbers reported exclude a small number (< 2%) of missing responses. Missing information also was negligible for most of the measures derived from the nurse survey, and ranged from roughly 2% to 5%.***
		(*d*) If applicable, describe analytical methods taking account of sampling strategy. ***Robust regression procedures were used to account for the clustering of patients and nurses in the hospitals and trusts.***
		(*e*) Describe any sensitivity analyses ***We report unadjusted and adjusted results in Tables 2 (p. 9) and 3 (p. 11), and found that our results remained significant even after adjustment for potential confounds.***
**Results**		
Participants	13*	(a) Report numbers of individuals at each stage of study—eg numbers potentially eligible, examined for eligibility, confirmed eligible, included in the study, completing follow-up, and analysed. ***The numbers of trusts, nurses and patients sampled are given in the abstract (p. ii) and on p. 3, where response rates are indicated).***
		(b) Give reasons for non-participation at each stage. ***We note on p. 4 that nurse survey data were available for only some 31 of the 161 trusts for which***
		(c) Consider use of a flow diagram. ***Not helpful here.***
Descriptive data	14*	(a) Give characteristics of study participants (eg demographic, clinical, social) and information on exposures and potential confounders. ***Numbers and characteristics of trusts and patients are shown in Tables 1 (p. 7), 2 (p. 9), and 3 (p. 11) (Also see related text). More detailed information on the trusts, patients and nurses in the study can be found in related papers that are cited.***
		(b) Indicate number of participants with missing data for each variable of interest.
Outcome data	15*	Report numbers of outcome events or summary measures. ***The patient satisfaction outcome data is given in the Appendix (separate from Main Document).***
Main results	16	(*a*) Give unadjusted estimates and, if applicable, confounder-adjusted estimates and their precision (eg, 95% confidence interval). Make clear which confounders were adjusted for and why they were included. ***Unadjusted and adjusted estimates are***

For peer review only - http://bmjopen.bmj.com/site/about/guidelines.xhtml

		shown in Tables 2 (p. 9) and 3 (p. 11).
		(*b*) Report category boundaries when continuous variables were categorized. ***Category boundaries for the categorized continuous variable, nurse work environment, are described on p. 6-7 of Methods.***
		(*c*) If relevant, consider translating estimates of relative risk into absolute risk for a meaningful time period. ***Not relevant.***
Other analyses	17	Report other analyses done—eg analyses of subgroups and interactions, and sensitivity analyses<br>***See 12b above.***
**Discussion**		
Key results	18	Summarise key results with reference to study objectives ***(see Discussion, p. 12-14) and Conclusion p. 14-15)***
Limitations	19	Discuss limitations of the study, taking into account sources of potential bias or imprecision. Discuss both direction and magnitude of any potential bias. ***See last paragraph in Discussion section on p. 14.***
Interpretation	20	Give a cautious overall interpretation of results considering objectives, limitations, multiplicity of analyses, results from similar studies, and other relevant evidence ***See Discussion (p. 12-14) and Conclusions sections (p. 14-15).***
Generalisability	21	Discuss the generalisability (external validity) of the study results. ***While point estimates of factors (like the nursing characteristics) and satisfaction likely vary from trust to trust, we believe the associations found are likely generalizable to all NHS trusts, as we note in the conclusion at the end (p. 14-15).***
**Other information**		
Funding	22	Give the source of funding and the role of the funders for the present study and, if applicable, for the original study on which the present article is based. ***Funding is from the European Union's Seventh Framework Program (223468) and the National Institute of Nursing Research, National Institutes of Health (R01NR014855). The funders had no role in the research or the writing of this manuscript.***

*Give information separately for exposed and unexposed groups.

Note: An Explanation and Elaboration article discusses each checklist item and gives methodological background and published examples of transparent reporting. The STROBE checklist is best used in conjunction with this article (freely available on the Web sites of PLoS Medicine at http://www.plosmedicine.org/, Annals of Internal Medicine at http://www.annals.org/, and Epidemiology at http://www.epidem.com/). Information on the STROBE Initiative is available at www.strobe-statement.org.

For peer review only - http://bmjopen.bmj.com/site/about/guidelines.xhtml
